# Theoretical insight into OH- and Cl-initiated oxidation of CF_3_OCH(CF_3_)_2_ and CF_3_OCF_2_CF_2_H & fate of CF_3_OC(X•)(CF_3_)_2_ and CF_3_OCF_2_CF_2_X• radicals (X=O, O_2_)

**DOI:** 10.1038/srep40264

**Published:** 2017-01-09

**Authors:** Feng-Yang Bai, Yuan Ma, Shuang Lv, Xiu-Mei Pan, Xiu-Juan Jia

**Affiliations:** 1 Institute of Functional Material Chemistry, National & Local United Engineering Lab for Power Battery, Faculty of Chemistry, Northeast Normal University, 130024 Changchun, People’s Republic of China; 2 School of Life Science, Northeast Normal University, 130024 Changchun, People’s Republic of China

## Abstract

In this study, the mechanistic and kinetic analysis for reactions of CF_3_OCH(CF_3_)_2_ and CF_3_OCF_2_CF_2_H with OH radicals and Cl atoms have been performed at the CCSD(T)//B3LYP/6-311++G(d,p) level. Kinetic isotope effects for reactions CF_3_OCH(CF_3_)_2_/CF_3_OCD(CF_3_)_2_ and CF_3_OCF_2_CF_2_H/CF_3_OCF_2_CF_2_D with OH and Cl were estimated so as to provide the theoretical estimation for future laboratory investigation. All rate constants, computed by canonical variational transition state theory (CVT) with the small-curvature tunneling correction (SCT), are in reasonable agreement with the limited experimental data. Standard enthalpies of formation for the species were also calculated. Atmospheric lifetime and global warming potentials (GWPs) of the reaction species were estimated, the large lifetimes and GWPs show that the environmental impact of them cannot be ignored. The organic nitrates can be produced by the further oxidation of CF_3_OC(•)(CF_3_)_2_ and CF_3_OCF_2_CF_2_• in the presence of O_2_ and NO. The subsequent decomposition pathways of CF_3_OC(O•)(CF_3_)_2_ and CF_3_OCF_2_CF_2_O**•** radicals were studied in detail. The derived Arrhenius expressions for the rate coefficients over 230–350 K are: *k*_T(1) _= 5.00 × 10^−24^*T*^3.57^ exp(−849.73/*T*), *k*_T(2) _= 1.79 × 10^−24^*T*^4.84^ exp(−4262.65/*T*), *k*_T(3) _= 1.94 × 10^−24^ *T*^4.18^ exp(−884.26/*T*), and *k*_T(4)_ = 9.44 × 10^−28^*T*^5.25^ exp(−913.45/*T*) cm^3^ molecule^−1^ s^−1^.

With people’s increasing awareness of environment protection, the issue of fine global warming potential (GWP) should be given more attention. Global warming will affect the viability and health of ecosystems, influencing shifts in the distribution of plants, animals, and even human settlements and health. Hydrofluorinated ethers (HFEs) have been considered as potential replacements to chlorofluorocarbons (CFCs) in many industrial applications^1^ for having no contribution to the stratospheric ozone depletion^2^,^3^. However, HFEs may have large GWP due to the presence of C–F bonds^4^. The HFEs can react with OH radicals and possibly with Cl atoms in the atomosphere^5^,^6^,^7^, so that they have a reduced effect on global warming. The oxidation of HFEs initiated by OH radicals and Cl atoms can lead to the formation of hydrofluorinated esters (FESs). Then, the FESs’ degradation can contribute to the environmental burden of trifluoroacetic acid (TFA) in troposphere^8^. In order to evaluate the environmental acceptability of HFEs and the possible contribution of the photo-oxidation of FESs to TFA formation or derivates in the environment, it is necessary to know the rate coefficients for the reactions of HFEs. The kinetics of reactions of HFEs with free radicals can aid our understanding of their environmental impacts and estimate their atmospheric lifetimes.

During these years, many theoretical^9^,^10^,^11^,^12^,^13^,^14^,^15^,^16^,^17^ and experimental^18^,^19^,^20^,^21^,^22^,^23^ investigations about the reactions of some volatile organic compounds (VOCs) with free radicals have been carried out in kinetic properties, reaction mechanisms, temperature dependence and more. Previous studies have generally evaluated the fates of HFEs at 298 ± 2 K only. First high-temperature calculations for the rate constants of the reactions of HFEs with OH radicals and Cl atoms are estimated to improve our knowledge of the combustion and atmospheric chemistry of HFEs. In this work, we have carried out a kinetic study of the CF_3_OCH(CF_3_)_2_ and CF_3_OCF_2_CF_2_H with OH radicals and Cl atoms, using density functional theory (DFT) and the dual-level direct dynamic method. As reported by Stevens *et al*.^24^. using the B3LYP/6-311 G(2d,2p) level, CF_3_OCF_2_CF_2_H has two stable conformers (defined as conformer a and b), within energy difference of 0.016 kcal/mol. Based on our calculation, the energy difference between the two conformers is only 0.059 kcal/mol at the B3LYP/6-311++G(d,p) level. The energy difference is so small that both of them may have contribution to the title reaction. In experiment, Andersen *et al*.^20^. reported reactions of CF_3_OCH(CF_3_)_2_ and CF_3_OCF_2_CF_2_H with OH radicals and Cl atoms through Fourier transform infrared (FTIR) smog chamber techniques at 296 ± 2 K. Subsequently, Wilson *et al*.^25^. investigated the kinetics of reaction CF_3_OCF_2_CF_2_H with OH by relative rate experiments. For reactions of CF_3_OCF_2_CF_2_H (b) + OH/Cl, Singh *et al*.^26^. have studied the mechanism and rate constants at only 296 K by conventional transition state theory (CTST). The calculated rate constants are close to the previous experimental work. Even so, little is known about the temperature dependencies and contributions ratios of two conformers of CF_3_OCF_2_CF_2_H from theory and experiment. In addition, the tunneling effects are found to be important for the HFEs reactions with free radicals, and canonical variational transition state theory (CVT) involving small curvature tunneling correction (SCT) can provide the relevant analysis for the tunneling and variational effects. Therefore, in the present work, high-level ab initio calculations and the dual-level direct dynamics method are performed on the mechanisms and kinetics of CF_3_OCH(CF_3_)_2_ + OH/Cl reactions and the dynamic properties of CF_3_OCF_2_CF_2_H + OH/Cl reactions.

Isotopic analysis allows us to understand the significant advances of atmospheric chemistry and global change. For instance, the air pollution, global warming, and climate change have been expounded by the study of the distribution of stable isotopes^27^. Kinetic isotope effects (KIEs) values can also provide useful information for explaining the stable isotope composition of atmospheric organic compounds^28^. In order to establish the isotopic effects in CF_3_OCH(CF_3_)_2_ and CF_3_OCF_2_CF_2_H and lay a direction for future experiments, KIEs for CF_3_OCH(CF_3_)_2_/CF_3_OCF_2_CF_2_H + OH/OD and CF_3_OCD(CF_3_)_2_/CF_3_OCF_2_CF_2_D + OH/Cl reactions are estimated firstly in theory. The temperature dependence of the H and D-abstraction rate constants of the title reactions in a wide temperature range are also discussed firstly.

In the atmosphere, the complex peroxy radicals can be produced by alkyl radical (CF_3_OC(•)(CF_3_)_2_ and CF_3_OCF_2_CF_2_**•**) reaction with O_2_. Then, the peroxy radicals are converted to the corresponding alkoxy radicals through reaction with NO, peroxy radicals, and other ways. For the reactions of peroxy radicals with NO, organic nitrates may also be formed in this cycle. Organic nitrates have been observed in ambient atmospheric aerosols and in secondary organic aerosols (SOA) formed from the oxidation of various volatile organic compounds in the presence of NO. The organic nitrates are relatively stable and hygroscopic and thus will play an important role in affecting the climate by acting as the components of aerosol organic matter. The above processes are recommended in experiment and theory as follows:


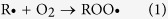



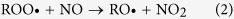



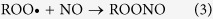



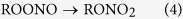


The alkoxy radical plays a significant role in the destruction of a variety of VOCs. Andersen *et al*.^20^. experimentally predicted the degradation mechanism and some products including CF_3_OC(O•)(CF_3_)_2_ and CF_3_OCF_2_CF_2_O**•** radicals. However, the information about the barrier heights for their subsequent degradation channels is not provided. Theoretical calculations can not only predict kinetic data in term of reaction barriers but also give the thermodynamic content of the products such as reaction energies and enthalpies. Our aim is to gain a further insight into the mechanism of the subsequent behaviors for CF_3_OC(O•)(CF_3_)_2_ and CF_3_OCF_2_CF_2_O**•** radicals. This is the first study to reveal the decomposition mechanisms of CF_3_OC(O•)(CF_3_)_2_ and CF_3_OCF_2_CF_2_O• in theory.

## Results and Discussion

### Electronic structures of stationary points

As for the prototypical species of HFEs, CF_3_OCH(CF_3_)_2_ and CF_3_OCF_2_CF_2_H can be attacked by OH radicals and Cl atoms by the following reaction channels:


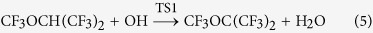



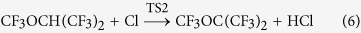



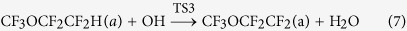



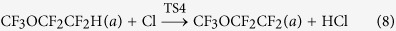



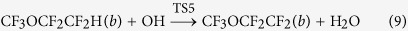



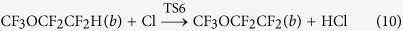


At the B3LYP/6-311++G(d,p) and M06-2X/6-311++G(d,p) levels, the optimized structural parameters of the reactants, products, complexes, and transition states for reactions of CF_3_OCH(CF_3_)_2 _+ OH/Cl and CF_3_OCF_2_CF_2_H (a, b) + OH/Cl are shown in Figure S1, along with the limited experimental bond distance and angle values^29^. It is seen that the optimized parameters of the stationary points are reasonable consistent with each other at the two levels. The theoretical bond distance of OH, H_2_O, HCl and bond angle in H_2_O are in good agreement with the corresponding experimental values. The vibrational frequencies of the stable points computed at the B3LYP/6-311++G(d,p) level are listed in Table S2, together with the experimental data of OH, H_2_O, and HCl^30^,^31^ The theoretical and experimental frequencies are in good agreement with each other within the largest deviation of 4.2%. The values of imaginary frequencies for TS1-TS6 are 1412*i*, 1101*i*, 958*i*, 848*i*, 949*i*, and 856*i* cm^−1^, respectively. Figure S1 demonstrates that the TS1 is reactant-like, and the reaction 5 may proceed via early transition state. However, the TS2 is product-like, and reaction 6 may proceed via late transition state. This phenomenon also appears in some similar species (CHF_2_OCF_2_CH_2_F, (CF_3_)_2_CHOCH_3_, (CF_3_)_2_CFOCH_3_, CF_3_CHFOCH_3_, CF_3_CHFOCF_3_, CHF_2_OCHFCF_3_, CF_3_OCH_2_CHF_2_, CF_3_OCHFCHF_2_)^8^,^9^,^12^−^16^ reaction with OH radicals or Cl atoms.

### Reactions mechanism and energetics

**Figure 1:**
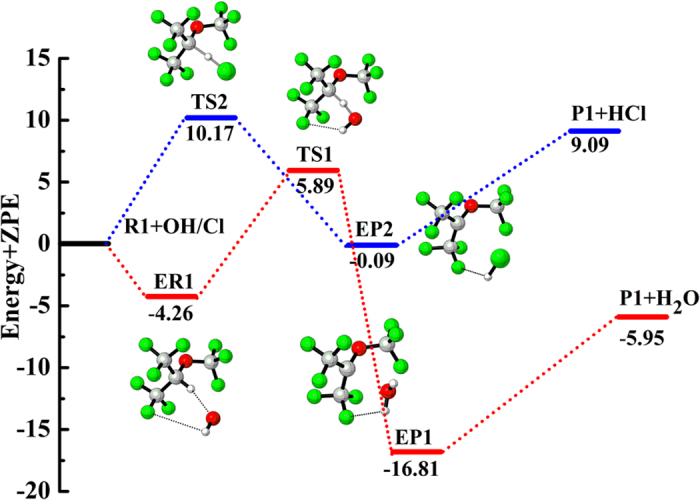
Schematic potential energy surface for reactions of CF_3_OCH(CF_3_)_2_ + OH/Cl. The relative energies (in kcal/mol) are calculated at the CCSD(T)//B3LYP/6-311++G(d,p) + ZPE level.

The schematic potential energy diagrams of CF_3_OCH(CF_3_)_2 _+ OH/Cl and CF_3_OCF_2_CF_2_H (a, b) + OH/Cl, obtained at the CCSD(T)//B3LYP/6-311++G(d,p) level, are plotted in Fig. 1 and Figure S2, respectively. For CF_3_OCF_2_CF_2_H (b) + OH/Cl, the calculated results are in line with the theoretical values reported by Singh *et al*.^26^. Therefore, we just discuss the reactions of CF_3_OCH(CF_3_)_2 _+ OH/Cl (reactions 5 and 6). For reaction 5, the relative energy of ER1 is lower than those of the reactants by 4.26 kcal/mol. Then, the corresponding product complex EP1 is produced with relative energies of 9.59 kcal/mol. The relative energy of transition state is calculated to be 5.89 kcal/mol. For reaction 6, product complex (EP2), with −0.09 kcal/mol energy, is formed via TS2 with the 10.17 kcal/mol potential barrier. Comparing the potential barriers of CF_3_OCH(CF_3_)_2_ + OH radicals with Cl atoms, we find that the barrier height of reaction with OH radical is lower than that with Cl atom. Thus, the hydrogen abstraction reaction rate constants of OH radical will be larger than that of Cl atom. Same conclusion can be found in similar reactions^14^,^15^,^26^.

Table 1 lists the reaction enthalpies (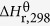), and reaction Gibbs free energies (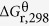) for reactions 5 and 6 at the B3LYP/6-311++G(d,p), and CCSD(T)//B3LYP/6-311++G(d,p) levels (kcal/mol) with the ZPE or TZPE corrections and the calculated bonds dissociation (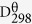) of the C–H in molecules CF_3_OCH(CF_3_)_2_ and CF_3_OCF_2_CF_2_H (a, b). The relative energies (Er) of main species for all reactions with ZPE corrections and *T*_1_ diagnostic values at CCSD(T)/6-311++G(d,p) level are listed in Table S3. The reaction enthalpies (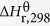), reaction Gibbs free energies (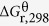) for reactions 7–10 are displayed in Table S4. Standard enthalpies of formation (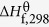) for the species at the B3LYP/6-311++G(d,p) and CCSD(T)//B3LYP/6-311++G(d,p) levels are listed in Table 2. As shown in Table S3, the *T*_1_ values of all species are within 0.006–0.017, indicating the multi-reference character in CCSD(T) wave functions may be ignored. Unless otherwise specified, energies used in the following discussion are at CCSD(T)//B3LYP/6-311++G(d,p) level. From Table 1, we can see that reaction of CF_3_OCH(CF_3_)_2_ with OH radical is exothermic with reaction enthalpy of −5.49 kcal/mol. However, reaction of CF_3_OCH(CF_3_)_2 _+ Cl is endothermic (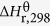 = 9.85 kcal/mol). For reaction of CF_3_OCH(CF_3_)_2_ with OH and Cl, the value of 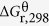 is −7.93 and 6.18 kcal/mol, respectively. The 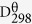 of C–H in molecule CF_3_OCH(CF_3_)_2_ is 113.96 kcal/mol, which is apparently higher than that of CF_3_OCF_2_CF_2_H. This can explain that the reactions of CF_3_OCH(CF_3_)_2_ + OH/Cl is hard to happen than CF_3_OCF_2_CF_2_H + OH/Cl. The following kinetic results will test this view. In summary, for CF_3_OCH(CF_3_)_2_, the reaction with OH radical is more favorable than its reaction with Cl atom in thermodynamics according to the reaction barrier heights and bonds dissociation energy. We also calculated enthalpies of formation (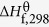) for the species CF_3_OCH(CF_3_)_2,_ CF_3_OC(CF_3_)_2_, CF_3_OCF_2_CF_2_H (a, b), and CF_3_OCF_2_CF_2_ (a, b) by the reactions 16–27. The experimental enthalpies of formation (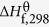) values^9^,^11^,^12^,^14^ involved in the reactions 16–27 are listed in Table S5. The enthalpies of formation (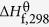) for the species CF_3_OCH(CF_3_)_2,_ CF_3_OC(CF_3_)_2,_ CF_3_OCF_2_CF_2_H (a), CF_3_OCF_2_CF_2_ (a), CF_3_OCF_2_CF_2_H (b), and CF_3_OCF_2_CF_2_ (b) are −525.61, −467.24, −412.89, −361.86, −412.88, and −361.70, respectively. Above content can enrich thermal chemical databases of CF_3_OCH(CF_3_)_2_ and CF_3_OCF_2_CF_2_H.

### Reaction path properties

Figure S3a–d show the changes of the bond lengths along the MEP as functions of *s* (amu)^1/2^bohr. For reactions 5, 6, 7, and 8, the changes are very similar, that is, the breaking bond (C–H) and the forming bond (O–H or Cl–H) change strongly, while the other bonds are few changes. Figure S4a–d depict the classical potential energy curve (*V*_MEP_), ground-state vibrational adiabatic energy curve (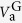), and zero-point energy curve (ZPE) as functions of *s* (amu)^1/2^bohr for the reactions 5, 6, 7, and 8, respectively. It can be seen that in Figure S4a–d, the plots of the 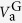 and *V*_MEP_ are similar in shape and locations, the curves of 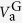 and *V*_MEP_ have the same zenith, indicating that the variational effect will be small in the calculation of the rate constant for the reactions 5, 6, 7, and 8. This will be borne out in the following study. The above conclusion about potential energy curves is similar with the previous theoretical work about the reactions of HFEs with OH radicals or Cl atoms^8^,^10^,^12^,^13^,^14^,^15^,^16^. The following study of the rate constants will also support this view deeply.

The variations of the generalized normal-mode vibrational frequencies along with the MEPs of reactions 5, 6, 7, and 8 are shown in Figure S5a–d, respectively. Figure S5a shows that in the reactant region at about *s *= −2.0 (amu)^1/2^bohr, the frequencies are associated with those of reactant complex; And in the product region at about 1.5 (amu)^1/2^bohr, the frequencies are associated with the product complex. It is obvious that the mode 1 in Figure S5a–d, to connect the C–H stretching vibration mode of CF_3_OCH(CF_3_)_2_ and CF_3_OCF_2_CF_2_H with the O–H (Cl–H) stretching vibration mode of H_2_O (HCl), drops significantly near about the saddle point. The dramatical changes are used to call “reaction mode”, which is known to be the typical behavior of H-transfer reactions.

### Kinetic rate constant calculations

**Figure 2:**
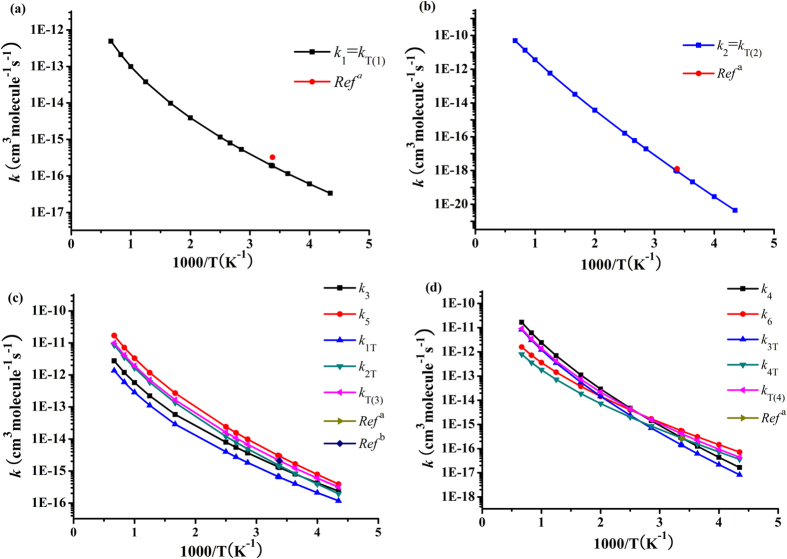
Computed individual rate constants (*k*_1_–*k*_6_, *k*_1T_–*k*_4T_) and total rate constants (*k*_T(1)_–*k*_T(4)_) for the title reactions together with the experimental data (^a^ref. 20.^b^ref. 25.) as functions of 1000/*T*.

Dual-level dynamics calculations are performed at the CCSD(T)//B3LYP/6-311++G(d,p) level to compute the reaction rate constants via the following reactions of 5–10. The CVT rate computations with the small-curvature tunneling (SCT) contributions are carried out for the six H-abstraction reactions in a wide temperature range from 230 to 1500 K. The TST, CVT, CVT/SCT rate constants of the reactions 5 (*k*_1_), 6 (*k*_2_), 7 (*k*_3_), and 8 (*k*_4_) are plotted in Figure S6a–d. The CVT/SCT rate constants of *k*_1_–*k*_6_ (for reactions 5–10), *k*_1T_–*k*_4T_, and the corresponding total rate constant *k*_T(1)_–*k*_T(4)_, along with the experimental values are depicted in Fig. 2a–d. Because of only one H-abstraction reaction channel for reaction of (CF_3_)_2_CHOCF_3_ + OH/Cl, the total rate constants *k*_T(1)_ = *k*_1_, *k*_T(2)_ = *k*_2._ The total rate constants *k*_T(3)_ (for the reaction of CF_3_OCF_2_CF_2_H with OH radical) and *k*_T(4)_ (for the reaction of CF_3_OCF_2_CF_2_H with Cl atom) can be obtained from the following expressions:


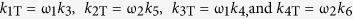



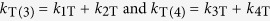


The ω_1_ and ω_2_ are the temperature dependent weight factors calculated from the Boltzmann population distribution for each conformer of CF_3_OCF_2_CF_2_H. The *k*_nT_ (n = 1–4) is corrected rate constant by weight factors for each reaction of CF_3_OCF_2_CF_2_H conformer with OH radicals or Cl atoms. From the Figure S6a–d, we can find that the variational effects, defined as the ratios of the TST and CVT rate constants, can be ignored over the whole temperature range of 230–1500 K for reactions 5, 6, 7, and 8. On the other hand, it is found that the tunneling effects, defined as the ratios of CVT/SCT and CVT rate constants, play an important role at the low temperatures for reactions 5, 6, 7, and 8. The tunneling effects should be taken into account at the temperature of 230–375 K for reaction 5, 230–375 K for reaction 7, and 230–1000 K for reaction 8. Taking reaction 8 as an example, the specific values of CVT/SCT and CVT rate constants are 152.29, 32.35, 4.77, and 1.61 at 230, 296, 500, and 1000 K, respectively. In the theoretical study on reactions of (CF_3_)_2_CFOCH_3_, CF_3_CHFOCF_3_, CHF_2_OCHFCF_3_^12^,^14^,^15^ with OH radicals and Cl atoms, similar tunneling effects can be found. Figure S6b shows that the CVT/SCT and CVT rate constants almost superposed on each other, implying that the tunneling contribution can be ignored for reaction 6.

Seen from Fig. 2a–d, for reactions of CF_3_OCH(CF_3_)_2_ + OH/Cl, the theoretical rate constants 1.88 × 10^−16^ and 9.17 × 10^−19^ cm^3^ molecule^−1^ s^−1^ are in good line with the corresponding experimental data 3.26 × 10^−16^ and 1.27 × 10^−18^ cm^3^ molecule^−1^ s^−1^ at 296 K^20^, respectively. It is pleasing to observe that the calculated rate constant, 2.11 × 10^−15^ cm^3^ molecule^−1^ s^−1^ of reaction CF_3_OCF_2_CF_2_H with OH radical at 296 ± 2 K, is in commendable accord with the experimental values of (2.26 ± 0.18) × 10^−15^ by Andersen *et al*.^20^. and 2.08 × 10^−15^ cm^3^ molecule^−1^ s^−1^ by Wilson *et al*.^25^. For reaction of CF_3_OCF_2_CF_2_H + Cl, theoretical overall rate constant (4.02 × 10^−16^ cm^3^ molecule^−1^ s^−1^) is in good agreement with the experimental value (2.70 × 10^−16^ cm^3^ molecule^−1^ s^−1^). The above good accordance between the experimental and theoretical result predicts the dynamic information is very credible. The positive temperature effect about the rate constants can be found for the reactions 5–10. This is not only the feature of the present reaction systems but also has been found in other similar reactions with OH radicals and Cl atoms by Devi *et al*.^9^. and other authors^8^,^10^,^11^,^12^,^13^,^14^,^15^,^16^.

### The contributions ratios

**Figure 3:**
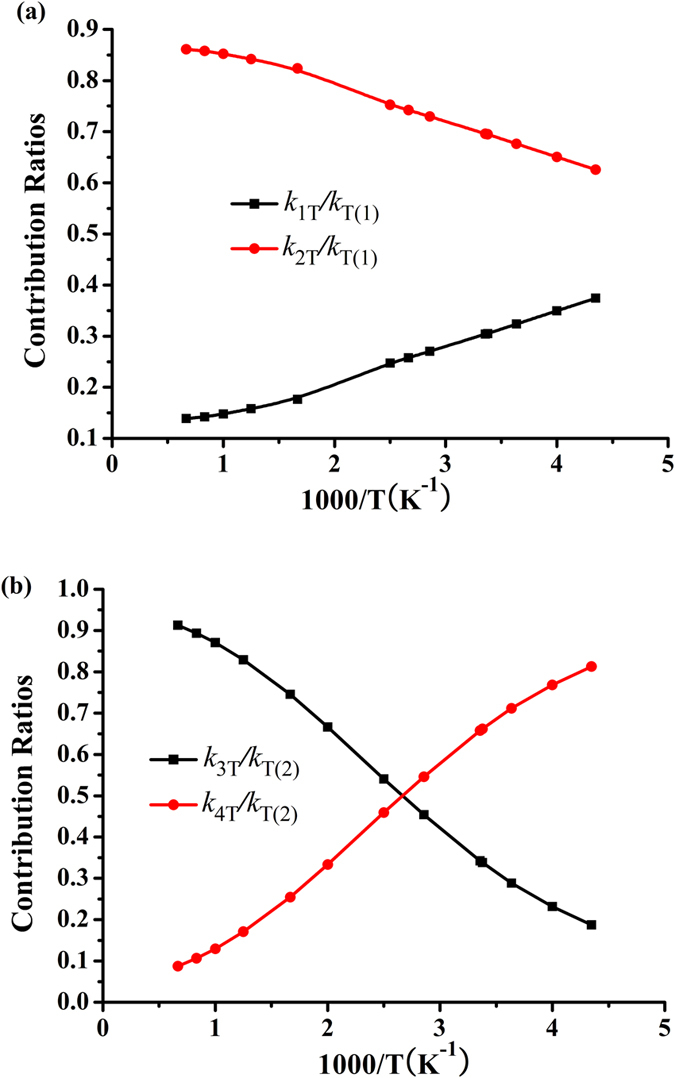
The contribution ratios as a function of 1000/*T* for reactions of CF_3_OCF_2_CF_2_H (**a,b**) + OH (**a**) and CF_3_OCF_2_CF_2_H (**a,b**) + Cl (**b**).

Figure 3a and b show the contributions of CF_3_OCF_2_CF_2_H (a) and CF_3_OCF_2_CF_2_H (b) to the reactions CF_3_OCF_2_CF_2_H + OH and CF_3_OCF_2_CF_2_H + Cl versus 1000/T between 230 and 1500 K, respectively. It can be seen from Fig. 3a that the contribution of CF_3_OCF_2_CF_2_H (a) to the overall reaction is less important than that of CF_3_OCF_2_CF_2_H (b) in the whole temperature range, and the contribution of CF_3_OCF_2_CF_2_H (b) increases with the increasing of the temperature. Figure 3b suggests that below 365 K, the contribution of CF_3_OCF_2_CF_2_H (b) to the overall reaction is more important than that of CF_3_OCF_2_CF_2_H (a). For example, the values of *k*_4T_/*k*_T(4)_ are 0.81 at 230 K and 0.55 at 350 K, respectively. However, as the temperature increases, CF_3_OCF_2_CF_2_H (a) prevails over CF_3_OCF_2_CF_2_H (b) and then becomes the major one, and the ratios of *k*_3T_/*k*_T(4)_ are 0.54 at 400 K and *k*_3T_/*k*_T(4)_ = 0.91 at 1500 K.

### Kinetic isotope effects (KIEs)

**Figure 4:**
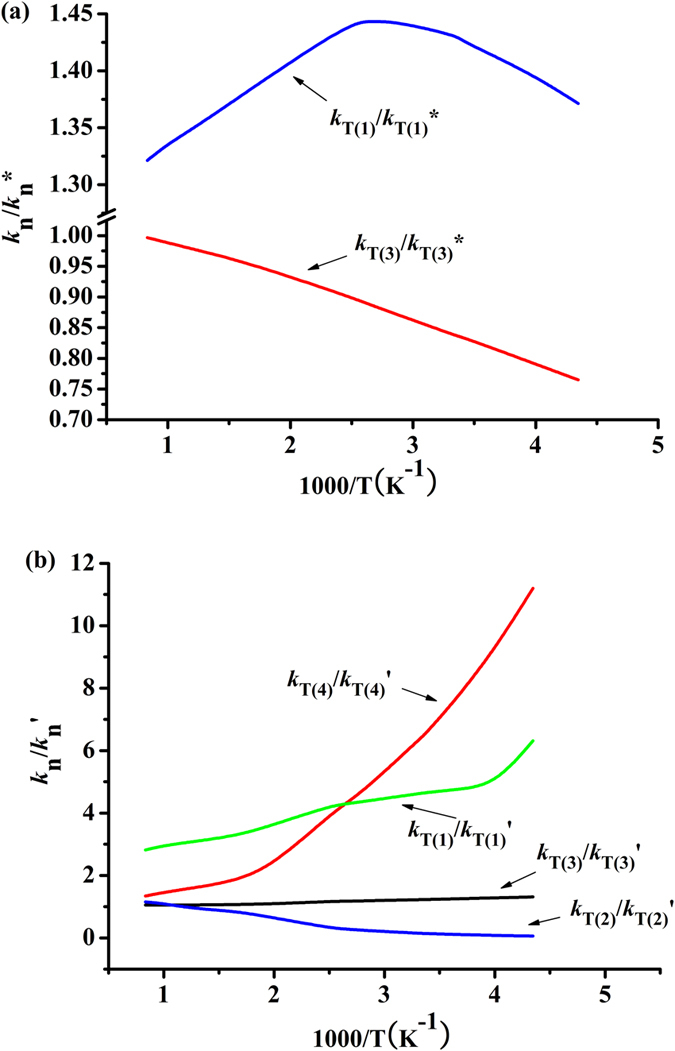
Plot of the calculated ratios versus 1000/*T* in the temperature range of 230–1500 K. The ratios of *k*_T(1)_/*k*_T(1)_*, *k*_T(3)_/*k*_T(3)_* in (**a**), and *k*_T(1)_/*k*_T(1)_’ *k*_T(2)_/*k*_T(2)_’, *k*_T(3)_/*k*_T(3)_’, *k*_T(4)_/*k*_T(4)_ ’ in (**b**).

The kinetic isotope effects (KIEs), defined as the ratios of between *k*^H^ and *k*^D^ for the reactions of CF_3_OCH(CF_3_)_2_/CF_3_OCF_2_CF_2_H + OD (R*) and CF_3_OCD(CF_3_)_2_/CF_3_OCF_2_CF_2_D + OH/Cl (R’) have been investigated over the whole temperature range. The calculated KIEs are displayed in the Fig. 4a and b. Figure 4a shows that the KIEs are smaller than 1 for reaction of (CF_3_)_2_CHOCF_3_ + OD, this is, in this reaction a noticeable inverse (*k*^H^/*k*^D^ < 1) exists. And the *k*_T(1)_/*k*_T(1)_* values increase with the increasing of temperature and the KIEs become negligible when the temperature is high. However, the KIEs of *k*_T(3)_/*k*_T(3)_* is larger than 1 (*k*^H^/*k*^D^ > 1) at the whole temperature range. The reaction of CF_3_OCD(CF_3_)_2_ + OH (*k*_1_’ = *k*_T(1)_’) comes to normal KIEs and the KIEs values decrease with increasing of temperature. The KIEs for *k*_2_’ (*k*_T(2)_’) are smaller than 1, and a noticeable inverse can be found. For the reaction of CF_3_OCF_2_CF_2_D + OH (*k*_T(3)_’), the values of KIEs are within 1.05–1.31 (around 1.0) in the whole temperature, indicating that the KIEs can be ignored. Note the KIEs of reaction of CF_3_OCF_2_CF_2_D + Cl (*k*_T(4)_’) need to consider because the values of KIEs are around to be 12. Comparing the KIEs of *k*’ with k*, we find that the results of KIEs for reactions of CF_3_OCH(CF_3_)_2_/CF_3_OCF_2_CF_2_H + OD (R*) and CF_3_OCD(CF_3_)_2_/CF_3_OCF_2_CF_2_D + OH (R’) are different. Taking a comparison between the reactions CF_3_OCH(CF_3_)_2_ + OD and CF_3_OCD(CF_3_)_2_ + OH, it is seen from Fig. 4a and b that the KIEs are different in temperature dependencies and a noticeable inverse or normal. Above isotope effects have also been found in many reactions^17^,^27^,^28^,^32^,^33^,^34^. Up to now, there is no corresponding experimental value, thus, our theoretical prediction can provide helpful information for further experimental study.

### Subsequent oxidation and decomposition pathways

**Figure 5:**
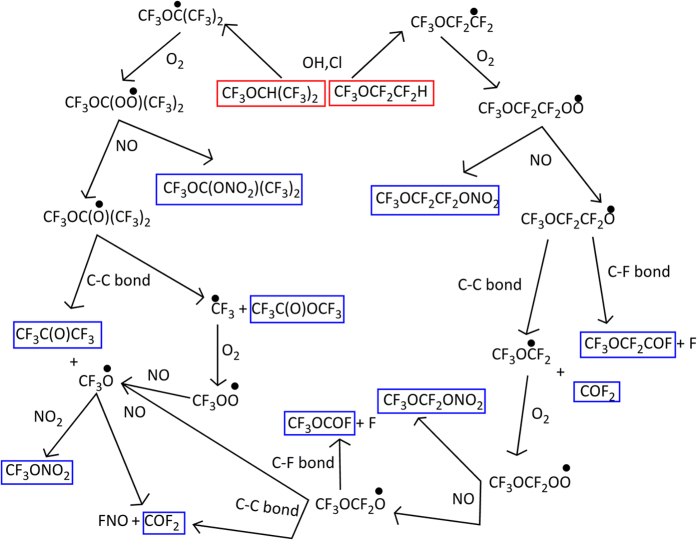
Atmospheric oxidation mechanism for CF_3_OCH(CF_3_)_2_ and CF_3_OCF_2_CF_2_H.

The mechanism and oxidation pathways of CF_3_OCH(CF_3_)_2_ and CF_3_OCF_2_CF_2_H to form the COF_2_ and CF_3_C(O)OCF_3_ is displayed in Fig. 5. From Fig. 5, the formed peroxy radicals (CF_3_OC(OO•)(CF_3_)_2_ and CF_3_OCF_2_CF_2_OO•) react with NO to form the organic nitrate and alkoxy radicals^35^. These processes are further refined and the geometries optimized at the B3LYP/6-311++G(d,p) level are listed in Figure S7. It is shown that the peroxynitrites were produced firstly and then it isomerizes to form organic nitrate or degrade to alkoxy radical and NO_2_. It can be seen from Figure S7a, CF_3_OC(OO•)(CF_3_)_2_ reacts with NO molecule to form IM1 (*t*-CF_3_OC(OONO)(CF_3_)_2_) and IM2 (*c*-CF_3_OC(OONO)(CF_3_)_2_) barrierlessly firstly. The IM1 can isomerize to IM2 via TSIM1-2 by overcoming 7.13 kcal/mol energy barrier. Then, IM1 can decompose into PIM1 (CF_3_OC(O•)(CF_3_)_2_ and NO_2_) via TSIM1-dec. The energy barrier for IM1 → CF_3_OC(O•)(CF_3_)_2_ + NO_2_ is 21.22 kcal/mol. Meanwhile, the IM2 can isomerize to IM3 (CF_3_OC(ONO_2_)(CF_3_)_2_) via TSIM2-iso by conquering a barrier of 7.01 kcal/mol. For the reaction of CF_3_OCF_2_CF_2_OO• with NO, we can find that the IM4 (*t*-CF_3_OCF_2_CF_2_OONO) and IM5 (*c*-CF_3_OCF_2_CF_2_OONO) are firstly formed with the relative energies of −23.07 and −24.25 kcal/mol, respectively. IM4 can also transform into IM5 through TSIM4–5 with an energy barrier of 9.26 kcal/mol. Afterwards, IM4 can dissociate into PIM4 (CF_3_OCF_2_CF_2_O• and NO_2_) through TSIM4-dec. The energy barrier for this dissociation process is 30.47 kcal/mol. However, the energy barrier for the process IM5 → TSIM5-iso → IM6 (CF_3_OCF_2_CF_2_ONO_2_) is 15.09 kcal/mol. Furthermore, the rate constant of IM1 decomposition is 2.75 × 10^−4^ s^−1^ at 298 K and ~10 order of magnitudes times smaller than that of isomerization pathway of IM2. In addition, the rate constant for IM4 dissociation is calculated to be 1.06 × 10^−9^ s^−1^ at 298 K, which is ~11 order of magnitudes times smaller than that of isomerization pathway of IM5. Therefore, we can get that the organic nitrate is more favorable to be produced than alkoxy radical. This result is in agreement with the investigation of RO_2_ reaction with NO (R=H, CH_3_, C_2_H_5_, n-C_3_H_7_, i-C_3_H_7_, 2-C_5_H_11_, and C_6_H_5_CHCH_2_OH) by Donahue *et al*.^36^ and An *et al*.^37^. The formed organic nitrates are relatively stable and thus the subsequent reactions from them will not be continued to discuss. We will pay our attention to the atmospheric behaviors of the alkoxy radicals. Atmospheric implications of organic nitrates will be discussed later.

The nature of the reaction mechanism for the unimolecular decomposition of CF_3_OC(O•)(CF_3_)_2_ and CF_3_OCF_2_CF_2_O• is carried out at the CCSD(T)//B3LYP/6-311++G(d,p) level of theory. For CF_3_OC(O•)(CF_3_)_2_, two possible channels are found in the subsequent reactions involved bond dissociation (C–C and C–O). Moreover, three possible processes included C–C/C–F bond dissociation and oxidation processes are listed as follows:


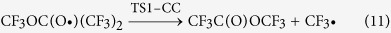



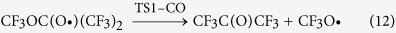



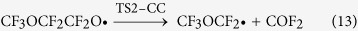



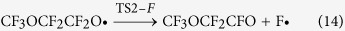



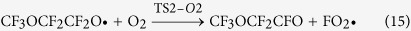


**Figure 6:**
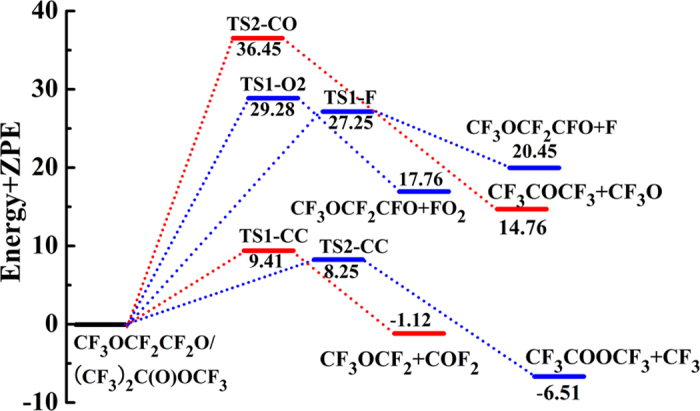
Potential energy profile (in kcal/mol) for the decomposition and oxidation pathways of CF_3_OC(O•)(CF_3_)_2_ and CF_3_OCF_2_CF_2_O• radicals at the CCSD(T)//B3LYP/6-311++G(d,p) level.

The optimized geometries and structural parameters of reactant and transition states for reactions 11–15 at the B3LYP level are depicted in Figure S8 in the Supporting Information. The thermodynamic data calculated at the CCSD(T)/6-311++G(d,p) and B3LYP/6-311++G(d,p) levels are summarized in Table 3 and the corresponding schematic potential energy surfaces are shown in Fig. 6. Transition vectors for all the transition states (TS1-CC, TS1-CO, TS2-CC, TS2-F, and TS2-O2) are obtained at 395*i*, 265*i*, 573*i*, 329*i*, and 414*i* cm^−1^, respectively, as recorded in Table S6 in the Supporting Information.

For reaction 11, the CF_3_OC(O•)(CF_3_)_2_ can proceed via TS1-CC to produce the hydrofluorinated esters of CF_3_C(O)OCF_3_ and CF_3_**•** radical by the C-C bond breakage. This process is exothermic (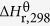 = −0.74 kcal/mol). From Figure S7, we can see that the C–O bond in TS1-CO will be broken. Table 3 shows that the process of bond breaking of C–O (reaction 12) bond is endothermic (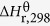 = 15.22 kcal/mol). Figure 6 shows the barrier heights of reactions 11 and 12 are 9.41 and 36.45 kcal/mol, respectively. This shows that the C–C bond fracture is more favorable than C–O bond fracture. These results are in good line with the experimental research^20^.

The CF_3_OCF_2_CF_2_O• can be dissociated into CF_3_OCF_2_• radical and COF_2_ by TS2-CC (reaction 13) with the C–C bond fracture. It is easy from Table 3 the C–C bond-cleavage pathway is significantly exothermic thus thermodynamically more facile. This process is accompanied by the energy barrier of 8.25 kcal/mol at the CCSD(T)//B3LYP/6-311++G(d,p) level. The transition state TS2-F corresponding to the C–F bond scission of CF_3_OCF_2_CF_2_O• (reaction 14) leading to F atom and CF_3_OCF_2_CFO is accompanied by an absorption of heat of 21.36 kcal/mol and thus is thermodynamically less probable. Table 3 and Fig. 6 suggest that the barriers of TS2-O2 are significantly higher than those of TS2-CC and TS2-F. Based on barrier heights, it may be concluded that thermal decomposition pathway of C–F bond (reaction 14) and oxidative pathway (reaction 15) not seem to vie with thermal decomposition pathway of C–C bond (reaction 13). In the end, ester CF_3_C(O)OCF_3_ may be the primary product for the subsequent decomposition pathways of CF_3_OC(O•)(CF_3_)_2_ radical. It can form CF_3_C(O)OH by hydrolysis^38^ and thus may contribute to the environmental burden of TFA. We can also summarize that the subsequent decomposition pathways of CF_3_OCF_2_CF_2_O• radical to produce CF_3_OCF_2_• radical and COF_2_. COF_2_ play an important role in producing CO_2_ and HF in the atmosphere and have potential to cause health or environmental problems. The above results are in good line with the experimental findings by Andersen *et al*.^20^.

As reported by Finlayson-Pitts *et al*.^39^, the reaction of alkoxy radical with NO_2_ is slow in the gas phase. Even at 100 ppb of NO_2_, the reaction rate constant for 3-alkoxy hexane with NO_2_ (75 s^−1^) cannot vie with its decomposition (~3 × 10^4^ s^−1^). Furthermore, the rate constants for the major decomposition pathways CF_3_OC(O•)(CF_3_)_2_ (reaction 11) and CF_3_OCF_2_CF_2_O• (reaction 13) are computed to be 3.0 × 10^9^ s^−1^ and 1.1 × 10^12^ s^−1^ at 298 K, respectively. The large decomposition rates may show that the decomposition reactions will precede their reactions with NO_2_ to form the corresponding nitrates. Thus, we predict that the alkoxy radicals of CF_3_OC(O•)(CF_3_)_2_ and CF_3_OCF_2_CF_2_O• may be unlikely reaction with NO_2_ to form CF_3_OC(ONO_2_)(CF_3_)_2_ and CF_3_OCF_2_CF_2_ONO_2_, rather than dissociate of themselves.

### Atmospheric Implications of CF_3_OCH(CF_3_)_2_ and CF_3_OCF_2_CF_2_H

The tropospheric lifetimes (τ) of CF_3_OCH(CF_3_)_2_ and CF_3_OCF_2_CF_2_H can be estimated by their removal reactions with OH radicals or Cl atoms. The atmospheric lifetimes can be shown as:


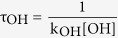



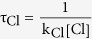



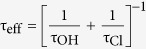


In the consideration of good agreement between the theoretically calculated and experimentally measured rate constants, we adopted the computed rate constants to estimate the lifetimes. Using the global atmospheric OH and Cl concentrations of 1 × 10^6^ and 1 × 10^3^ molecule cm^−3^ 5,7, the lifetimes of CF_3_OCH(CF_3_)_2_ and CF_3_OCF_2_CF_2_H are estimated to be 168 and 15 years at 296 K. The estimated lifetimes are respectively in good agreement with the experimental values (estimated based on the experimental rate constants) 117.0 and 16.9 years (18.8 years)^40^,^41^. These relatively long times suggest that in the atmosphere CF_3_OCH(CF_3_)_2_ and CF_3_OCF_2_CF_2_H are environmentally unfriendly.

In order to further assess their atmospheric implications, the global warming potentials (GWPs)^42^,^43^ to carbon dioxide of CF_3_OCH(CF_3_)_2_ and CF_3_OCF_2_CF_2_H are computed at different time horizons of 20, 100, and 500 years. We calculated the GWPs for CF_3_OCH(CF_3_)_2_ and CF_3_OCF_2_CF_2_H by the following formula:


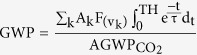


Where (*A*_k_) is the intensities of the corresponding vibrational mode *k.* 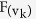
is the radiative forcing function per unit cross section per wave number (W m^−2^ (cm^−1^)^−1^ (cm^2^ molecule^−1^)^−1^) evaluated at the scaled band center frequency (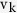)^43^ TH is the time horizon. 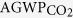
is the absolute global warming potential for carbon dioxide^44^. The radiative efficiencies (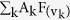) are 0.632 and 0.635 W m^−2^ ppb^−1^ for CF_3_OCH(CF_3_)_2_ and CF_3_OCF_2_CF_2_H, respectively, by using the method proposed by Pinnock *et al*.^43^. The underlying data of frequencies, IR intensities, and radiative forcing used in calculation of GWP of CF_3_OCH(CF_3_)_2_ and CF_3_OCF_2_CF_2_H are presented in Table S7 and S8, respectively. For compounds that are not well-mixed in the atmosphere, the correction factors, *f(τ*), based on the lifetime of the compounds are needed. The corresponding expression can be shown as in the Hodnebrog *et al*.’s work^45^. The *f(τ*) values for CF_3_OCH(CF_3_)_2_ and CF_3_OCF_2_CF_2_H are calculated to be 0.991 and 0.966, respectively. Herein, the lifetimes corrected radiative efficiencies are 0.626 W m^−2^ ppb^−1^ CF_3_OCH(CF_3_)_2_ and 0.613 W m^−2^ ppb^−1^ for CF_3_OCF_2_CF_2_H. The corresponding GWPs values are displayed in Table 4. We can see for Table 4 that the GWPs of CF_3_OCH(CF_3_)_2_ and CF_3_OCF_2_CF_2_H at 20 years of time horizon are 9371 and 6877, respectively. These large values show that the contribution of two HFEs to GWP will be significant.

With regard to the atmospheric implications of CF_3_OCH(CF_3_)_2_ and CF_3_OCF_2_CF_2_H, the following statements can be taken into account: (1) CF_3_OCH(CF_3_)_2_ and CF_3_OCF_2_CF_2_H will not contribute to stratospheric ozone depletion. (2) It is noteworthy that the atmospheric lifetimes of CF_3_OCH(CF_3_)_2_ and CF_3_OCF_2_CF_2_H are approximately 168 and 15 years, respectively, and consequently they have remarkable GWP (see above). (3) The atmospheric oxidation of CF_3_OCH(CF_3_)_2_ and CF_3_OCF_2_CF_2_H gives organic nitrates (CF_3_ONO_2_, CF_3_OCF_2_NO_2_, CF_3_OCF_2_CF_2_NO_2_, (CF_3_)_2_C(ONO_2_)OCF_3_), CF_3_OCOF, CF_3_COOCF_3_, COF_2_, and CF_3_COCF_3_. The organic nitrates are hygroscopic, and thus they influence the climate by serving as cloud condensation nuclei and components of aerosol organic matter. COF_2_ is removed from the troposphere via contact with water surfaces and hydrolysis to HF and CO_2_ in atmosphere. The CF_3_COF, CF_3_COOCF_3_, and CF_3_COCF_3_ can be hydrolysis to produce TFA and HF. All in all, the contributing oxidation products may increase the acidity of precipitation and consequently they have negative impact on the air quality.

The three-parameter Arrhennius fits based on the calculated rate constants (in units of cm^3^ molecule^−1^ s^−1^) at the CCSD(T)//B3LYP/6-311++G(d,p) level within 230–350 K for the main emphasis is on atmospheric applications give expressions as follows:


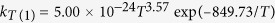



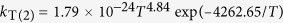



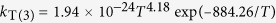



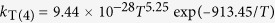


## Discussion and Summary

DFT methods are adopted to investigate kinetic contents of reactions of CF_3_OCH(CF_3_)_2_ and CF_3_OCF_2_CF_2_H with OH radical and Cl atom. The high-level energies for the stationary points are refined at the CCSD(T)//B3LYP/6-311++G(d,p) level. All the H-abstraction reactions rate constants are calculated by CVT combined with SCT correction. The agreement between theoretical and experiment result is very excellent. In the temperature range of 230–1500 K, the variational effect is almost not existing or almost negligible. The tunneling effect is significant in the whole lower temperatures for reactions 5, 7, and 8. All the rate constants have positive temperature effects. Calculated results show that two conformers of CF_3_OCF_2_CF_2_H are both important for total rate constant of reactions of CF_3_OCF_2_CF_2_H + OH/Cl.

In addition, kinetic isotope effects for the reactions CF_3_OCH(CF_3_)_2_/CF_3_OCF_2_CF_2_H + OD (R*) and CF_3_OCD(CF_3_)_2_/CF_3_OCF_2_CF_2_D + OH/Cl (R’) have been investigated. The atmospheric lifetimes of (CF_3_)_2_CHOCF_3_ and CF_3_OCF_2_CF_2_H are estimated to be 168 and 15 years, respectively. The GWP_(20 years)_ values are calculated to be 9371 and 6877, respectively, which show that their contributions to the global warming are significant and should be given more concerns. The peroxy radicals of CF_3_OC(OO•)(CF_3_)_2_ and CF_3_OCF_2_CF_2_OO• formed by the alkyl radicals with O_2_ can produce the CF_3_OC(ONO_2_)(CF_3_)_2_ and CF_3_OCF_2_CF_2_ONO_2_ in the NO-rich environment. The CF_3_OC(ONO_2_)(CF_3_)_2_ and CF_3_OCF_2_CF_2_ONO_2_ are thermally stable nitrate and thus will play a very important role in SOA formation. The subsequent reaction pathways of CF_3_OC(O•)(CF_3_)_2_ and CF_3_OCF_2_CF_2_O• radicals in the gas phase are also investigated in detail. The primary degradation channels of CF_3_OC(O•)(CF_3_)_2_ and CF_3_OCF_2_CF_2_O• radicals are the C–C bond cleavage to form the COF_2_ and CF_3_C(O)OCF_3_, respectively, which is in consistent with the experimental findings. The high toxic excitant gas COF_2_ can be hydrolysis into CO_2_ and HF which can increase the acidity of precipitation. The CF_3_C(O)OCF_3_ can be transformed into highly soluble compounds TFA and HF which may be rapidly incorporated into cloud droplets, contributing to the acidity of precipitation. The standard enthalpies of formation for the species are also calculated. The three-parameter Arrhenius expressions for the title reactions are *k*_T(1) _= 5.00 × 10^−24^*T*^3.57^ exp(−849.73/*T*), *k*_T(2) _= 1.79 × 10^−24^*T*^4.84^ exp(−4262.65/*T*), *k*_T(3) _= 1.94 × 10^−24^ *T*^4.18^ exp(−884.26/*T*), and *k*_T(4) _= 9.44 × 10^−28^*T*^5.25^ exp(−913.45/*T*) cm^3^ molecule^−1^ s^−1^. We hope our theoretical studies may provide a good estimate for further laboratory understanding the relevant chemical reactions.

## Methodology

### Electronic structure calculations

The equilibrium geometries of the reactants, products, complexes, and transition states are optimized at the B3LYP^46^,^47^,^48^ of theory conjunct with the standard 6-311++G(d,p) basis set using the Gaussian 09 package^49^. The B3LYP method is more widely used for predicting the reliable geometries of the stationary points, and found to be accurate from the previous studies^12^,^15^,^16^,^50^,^51^,^52^,^53^,^54^,^55^. Moreover, B3LYP theory is able to suppress effectively the problem of spin contamination. All the transition states are verified by the intrinsic reaction coordinate (IRC) calculations to connect the corresponding reactants to products at the B3LYP/6-311++G(d,p) level. Geometries of stationary points are also optimized at the M06-2X/6-311++G(d,p)^56^ level to ensure the accuracy of data. In order to obtain more reliable relative energy, single points energy calculations have been performed by the method of CCSD(T)/6-311++G(d,p)^57^,^58^. The differences of energies for these transition states between the CCSD(T)//B3LYP/6-311++G(d,p) and CCSD(T)//M06-2X/6-311++G(d,p) levels are within 0.07–0.49 kcal/mol (See Table S1), suggesting that the B3LYP level is reasonable and applicable in the current system. As also reported in the previous work for the HFEs with radicals^11^, the single points energies obtained at the CCSD(T) level based on the M06-2X geometries are very close to the results based on the B3LYP geometries (within the largest difference of 0.40 kcal/mol), thus, we just discuss the single points energy at the CCSD(T)//B3LYP/6-311++G(d,p) level in this paper. The *T*_1_ values of all species are checked to guarantee that CCSD(T) wave function is not affected by multi-reference character.

### Thermodynamics and Kinetics

The enthalpies of formation (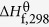) are the important thermodynamic parameters which can be estimated by using isodesmic reactions. Herein, we predict the values of 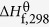 for CF_3_OCH(CF_3_)_2_, CF_3_OC(CF_3_)_2_, CF_3_OCF_2_CF_2_H (a, b), and CF_3_OCF_2_CF_2_ (a, b) by using the following reactions:


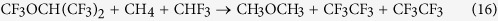



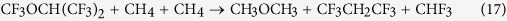



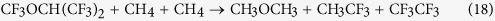



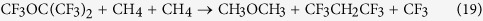



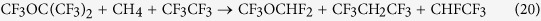



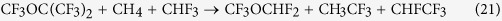



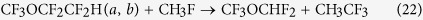



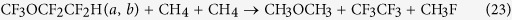



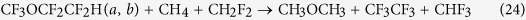



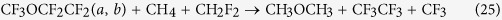



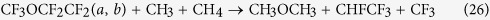



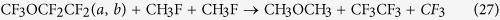


The kinetic rate constants, over the temperature range of 230–1500 K, are computed using the canonical variational transition state theory (CVT)^59^,^60^ involving small curvature tunneling correction (SCT)^61^,^62^ through the POLYRATE-Version 9.7 computer program^63^. Dynamic computations are performed using the values obtained from the CCSD(T)//B3LYP/6-311++G(d,p) level to make a direct comparison to the previous investigations^9^,^11^,^12^,^15^,^16^. The relevant expressions are given as:


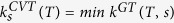



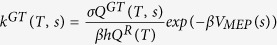


In above expressions, *s* is the location of the generalized transition state on the IRC; *σ* is the symmetry factor; _β_ equals (*k*_B_*T*)^−1^ where *k*_B_ is Boltzmann’s constant; *h* is Planck’s constant; The *κ* is the tunneling factor; and *Q*^*GT*^ and *Q*_*R*_ are partition functions for the generalized transition state and reactants, respectively. In the calculations of the electronic partition functions, two electronic states for OH radical, with a 140 cm^−1^ splitting in the ^2^Π_1/2_ and ^2^Π_3/2_ ground states, are included. The two low-lying electronic states, ^2^P_1/2_ and ^2^P_3/2_, with a splitting of 881 cm^−1^ of Cl atom, are also included. For the title reactions of 5 and 7–10, there are weak hydrogen-bonded complexes formed at the entrance channels. Hence, the stepwise mechanism occurs, that is, the first reversible step leads to the formation of reactant complex, and the second irreversible step yields the corresponding products. For this case, we use “well” option to think about the contributions of reactant complex in the Polyrate suite. This method has been applied successfully to describe the reactions of the free radical with several volatile organic chemicals^14^,^15^,^16^,^64^,^65^,^66^. Detailed discussions can be shown in Alvarez-Idaboy *et al*.’s works^64^,^65^,^66^.

## Additional Information

**How to cite this article**: Bai, F.-Y. *et al*. Theoretical insight into OH- and Cl-initiated oxidation of CF_3_OCH(CF_3_)_2_ and CF_3_OCF_2_CF_2_H & fate of CF_3_OC(X•)(CF_3_)_2_ and CF_3_OCF_2_CF_2_X• radicals (X=O, O_2_). *Sci. Rep.* **7**, 40264; doi: 10.1038/srep40264 (2017).

**Publisher's note:** Springer Nature remains neutral with regard to jurisdictional claims in published maps and institutional affiliations.

## Supplementary Material

Supporting Information

## Figures and Tables

**Table 1:** Reaction enthalpies (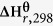) and reaction Gibbs free energies (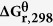) for reactions 5 and 6 at the B3LYP/6-311++G(d,p), and CCSD(T)//B3LYP/6-311++G(d,p) levels (kcal/mol) and the calculated bonds dissociation energy (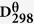) of the C–H in molecules CF_3_OCH(CF_3_)_2_ and CF_3_OCF_2_CF_2_H (a,b).

	B3LYP	CCSD(T)//B3LYP
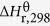		
CF_3_OCH(CF_3_)_2_ + OH → (CF_3_)_2_COCF_3_ + H_2_O	−15.73	−5.49
CF_3_OCH(CF_3_)_2_ + Cl → (CF_3_)_2_CHOCF_3_ + HCl	−1.12	9.85
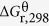		
CF_3_OCH(CF_3_)_2_ + OH → (CF_3_)_2_COCF_3_ + H_2_O	−18.17	−7.93
CF_3_OCH(CF_3_)_2_ + Cl → (CF_3_)_2_CHOCF_3_ + HCl	−4.79	6.18
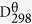		
CF_3_OCH(CF_3_)_2_→(CF_3_)_2_COCF_3 _+ H	105.98	113.96
CF_3_OCF_2_CF_2_H (a)→CF_3_OCF_2_CF_2_(a) + H	107.09	107.80
CF_3_OCF_2_CF_2_H (b)→CF_3_OCF_2_CF_2_(b) + H	107.07	107.95

**Table 2:** Standard enthalpies of formation ( 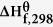 ) (in kcal/mol) for the species CF_3_OCH(CF_3_)_2_, CF_3_OC(CF_3_)_2_ CF_3_OCF_2_CF_2_H(a,b), and CF_3_OCF_2_CF_2_(a,b) at the B3LYP/6-311++G(d,p) and CCSD(T)//B3LYP/6-311++G(d,p) levels.

Species	Reactions	B3LYP		CCSD(T)//B3LYP	
		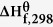	average	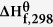	average
	16	−523.16		−526.27	
CF_3_OCH(CF_3_)_2_	17	−520.20	−520.95	−525.86	−525.61
	18	−519.48		−524.70	
	19	−470.83		−468.23	
CF_3_OC(CF_3_)_2_	20	−468.55	−469.07	−467.33	−467.24
	21	−467.84		−466.16	
	22	−411.28		−412.59	
CF_3_OCF_2_CF_2_H (a)	23	−410.27	−410.61	−412.58	−412.89
	24	−410.27		−413.51	
	25	−361.95		−361.86	
CF_3_OCF_2_CF_2_ (a)	26	−355.45	−360.14	−361.35	−361.86
	27	−363.01		−362.38	
	22	−411.27		−412.58	
CF_3_OCF_2_CF_2_H (b)	23	−410.26	−411.40	−412.57	−412.88
	24	−412.66		−413.50	
	25	−362.02		−361.70	
CF_3_OCF_2_CF_2_ (b)	26	−355.51	−360.20	−361.19	−361.70
	27	−363.08		−362.22	

**Table 3:** Computed barrier heights ( 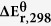 ), enthalpies ( 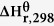 ), and Gibbs free energies ( 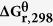 ) for reactions involved in thermal decomposition of the alkoxy radicals at B3LYP/6-311++G(d,p) and CCSD(T)//B3LYP/6-311++G(d,p) levels of theory.

	B3LYP/6-311++G(d,p)			CCSD(T)//B3LYP/6-311++G(d,p)		
Reactions	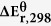	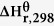	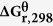	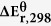	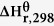	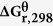
11	5.09	−7.95	−14.55	9.41	−0.74	−7.34
12	20.24	9.52	−4.50	36.45	15.22	1.19
13	2.78	−12.87	−26.66	8.25	−6.11	−19.89
14	24.35	24.21	14.80	27.25	21.36	11.95
15	26.60	13.76	10.78	29.28	17.40	14.42

**Table 4:** Atmospheric lifetimes (τ_eff_) of CF_3_OCH(CF_3_)_2_ and CF_3_OCF_2_CF_2_H (in years) at 296 K and global warming potentials (GWPs) computed for the time horizons of 20, 100, and 500 years using the B3LYP/6-311++G(d,p) level of theory.

Molecules	Atmospheric lifetime (years)				GWPs				
					20		100		500
CF_3_OCH(CF_3_)_2_	168	216^a^	117.0^b^		9371	6680^a^	11470	8230^a^	7596
CF_3_OCF_2_CF_2_H	15	27^a^	16.9^b^	18.8^c^	6877	6740^a^	2875	3690^a^	899

^a^Obtained from ref. 20.

^b^Obtained from ref. 40.

^c^Obtained from ref. 41.
