# Supplementary material for: Theoretical insight into OH- and Cl-initiated oxidation of CF3OCH(CF3)2 and CF3OCF2CF2H & fate of CF3OC(X•)(CF3)2 and CF3OCF2CF2X• radicals (X=O, O2)
Source: Sci Rep. 2017 Jan 9;7:40264. doi: 10.1038/srep40264 (PMC5220334; doi:10.1038/srep40264)
Supplement: Supporting Information [file srep40264-s1.doc]

**Supplementary Information:**

**Theoretical insight into OH- and Cl-initiated oxidation of CF_3_OCH(CF_3_)_2_ and CF_3_OCF_2_CF_2_H & fate of CF_3_OC(X•)(CF_3_)_2_ and CF_3_OCF_2_CF_2_X• radicals (X=O, O_2_)**

Feng-Yang Bai,^a^ Yuan Ma,^a^ Shuang Lv,^a^ Xiu-Mei Pan*^a^ and Xiu-Juan Jia,*^b^

^a^ Institute of Functional Material Chemistry, National & Local United Engineering Lab for Power Battery, Faculty of Chemistry, Northeast Normal University, 130024 Changchun, People’s Republic of China

^b^ School of Life Science, Northeast Normal University, 130024 Changchun, People’s Republic of China

* Corresponding author: Prof. Xiu-Mei Pan

Fax: +86-431-85099511;

Tel: +86-431-85099291;

E-mail address: panxm460@nenu.edu.cn

**Table S1**. Relative energies (Er) based on the corresponding reactants for the transition states TS1–TS6 obtained at the CCSD(T)//B3LYP/6-311++G(d,p) and CCSD(T)//M06-2X/6-311++G(d,p) levels (in kcal/mol).

| Transition States | CCSD(T)//B3LYP/6-311++G(d,p) | CCSD(T)//M06-2X/6-311++G(d,p) |
| --- | --- | --- |
| TS1 | 5.89 | 5.84 |
| TS2 | 10.17 | 9.68 |
| TS3 | 5.97 | 6.12 |
| TS4 | 8.29 | 7.97 |
| TS5 | 5.43 | 5.50 |
| TS6 | 7.94 | 7.70 |

**Table S2.** Calculated frequencies (Experimental values in parenthesis) (in cm^-1^) and <*s^2^*> values for the reactants, products, transition states, and complexes for the title reactions at the B3LYP/6-311++G(d,p) level.

| Species | B3LYP/6-311++G(d,p) | <*s*^2^> |
| --- | --- | --- |
| (CF_3_)_2_CHOCF_3_ | 17, 31, 58, 73, 143, 160, 209, 220, 288, 308, 315, 339, 450, 472, 517, 530, 544, 547, 608, 648, 667, 683, 737, 862, 894, 898, 1093, 1112, 1130, 1165, 1178, 1199, 1230, 1245, 1265, 1293, 1369, 1406, 3104 | 0.0 |
| CF_3_OCF_2_CF_2_H (a) | 34, 63, 86, 131, 201, 216, 334, 345, 363, 450, 519, 544, 560, 585, 627, 644, 754, 820, 956, 1099, 1112, 1125, 1135, 1196, 1214, 1248, 1294, 1367, 1424, 3103 | 0.0 |
| CF_3_OCF_2_CF_2_H (b) | 36, 58, 84, 129, 206, 249, 312, 340, 354, 423, 505, 530, 557, 606, 635, 674, 783, 843, 899, 1097, 1121, 1132, 1139, 1196, 1210, 1232, 1309, 1366, 1417, 3110 | 0.0 |
| (CF_3_)_2_COCF_3_ | 5, 35, 47, 57, 87, 147, 194, 225, 292, 311, 312, 341, 448, 458, 496, 533, 539, 548, 602, 613, 659, 687, 706, 768, 879, 973, 1091, 1116, 1133, 1137, 1189, 1196, 1217, 1263, 1319, 1381 | 0.754 |
| CF_3_OCF_2_CF_2_ (a) | 34, 52, 81, 123, 194, 214, 336, 345, 363, 452, 517, 544, 558, 586, 633, 650, 730, 817, 905, 1077, 1108, 1144, 1196, 1209, 1250, 1265, 1383 | 0.752 |
| CF_3_OCF_2_CF_2_ (b) | 40, 52, 82, 129, 206, 226, 316, 340, 354, 425, 503, 529, 577, 603, 635, 680, 729, 825, 893, 1054, 1120, 1148, 1195, 1221, 1251, 1265, 1382 | 0.752 |
| OH | 3709 (3775) | 0.752 |
| HCl | 2927 (2991) | 0.0 |
| H_2_O | 1602 (1595) , 3818 (3657), 3923 (3756) | 0.0 |
| TS1 | 1412*i*, 22, 32, 51, 62, 83, 92, 131, 136, 157, 209, 214, 288, 310, 314, 339, 385, 445, 498, 538, 531, 546, 547, 603, 659, 678, 685, 740, 766, 879, 897, 937, 1054, 1137, 1149, 1166, 1171, 1192, 1196, 1226, 1253, 1280, 1284, 1455, 3737 | 0.757 |
| TS2 | 1101*i*, 21, 32, 44, 55, 71, 74, 121, 153, 197, 206, 284, 300, 307, 316, 338, 438, 480, 527, 530, 543, 544, 597, 650, 672, 686, 751, 797, 836, 890, 973, 976, 1120, 1127, 1164, 1188, 1193, 1202, 1236, 1259, 1264, 1292 | 0.756 |
| TS3 | 958*i*, 36, 41, 52, 83, 90, 128, 142, 201, 217, 336, 345, 363, 449, 467, 524, 548, 576, 623, 631, 667, 739, 816, 842, 979, 1091, 1125, 1142, 1177, 1188, 1206, 1240, 1250, 1330, 1410, 3736 | 0.756 |
| TS4 | 848*i*, 31, 38, 49, 83, 115, 136, 200, 215, 298, 336, 345, 362, 450, 521, 548, 579, 621, 631, 663, 811, 821, 873, 889, 993, 1093, 1147, 1159, 1203, 1215, 1252, 1256, 1331 | 0.755 |
| TS5 | 949*i*, 37, 41, 66, 80, 113, 136, 146, 208, 253, 311, 339, 354, 420, 479, 520, 544, 579, 633, 640, 679, 738, 815, 881, 937, 1101, 1121, 1144, 1181, 1198, 1210, 1225, 1256, 1331, 1416, 3737 | 0.756 |
| TS6 | 856*i*, 24, 33, 59, 75, 115, 133, 207, 232, 308, 322, 346, 364, 428, 516, 535, 577, 622, 637, 678, 815, 857, 873, 889, 961, 1095, 1136, 1166, 1201, 1230, 1253, 1256, 1331 | 0.755 |
| ER1 | 10, 20, 26, 34, 60, 69, 70, 143, 159, 180, 192, 209, 219, 289, 308, 316, 339, 450, 472, 517, 530, 545, 547, 609, 648, 667, 683, 736, 861, 895, 897, 1091, 1107, 1126, 1165, 1179, 1194, 1228, 1249, 1258, 1293, 1378, 1414, 3084, 3701 | 0.752 |
| ER3 | 13, 21, 39, 62, 82, 88, 93, 132, 192, 201, 216, 335, 344, 363, 450, 519, 543, 559, 586, 627, 644, 753, 819, 955, 1099, 1106, 1119, 1129, 1192, 1216, 1245, 1295, 1384, 1436, 3122, 3708 | 0.752 |
| ER4 | 8, 10, 22, 39, 68, 88, 131, 201, 217, 335, 345, 363, 450, 519, 544, 560, 585, 627, 644, 754, 820, 956, 1099, 1112, 1125, 1135, 1196, 1215, 1248, 1295, 1372, 1426, 3105 | 0.752 |
| ER5 | 9, 20, 40, 56, 65, 86, 130, 158, 183, 207, 251, 312, 340, 355, 424, 504, 529, 557, 606, 635, 674, 783, 843, 898, 1095, 1113, 1131, 1135, 1195, 1209, 1227, 1312, 1372, 1420, 3117, 3704 | 0.752 |
| ER6 | 12, 13, 34, 39, 57, 86, 130, 206, 249, 311, 340, 354, 424, 504, 530, 557, 606, 635, 674, 783, 843, 898, 1096, 1120, 1132, 1137, 1196, 1210, 1231, 1310, 1373, 1419, 3115 | 0.752 |
| EP1 | 15, 37, 43, 54, 61, 68, 86, 107, 125, 143, 167, 194, 220, 222, 289, 311, 317, 343, 445, 462, 503, 531, 540, 546, 601, 619, 665, 693, 707, 772, 879, 965, 1080, 1109, 1124, 1138, 1190, 1205, 1220, 1244, 1321, 1372, 1614, 3813, 3917 | 0.753 |
| EP2 | 19, 22, 27, 31, 41, 48, 60, 92, 101, 114, 140, 195, 224, 288, 310, 315, 342, 443, 460, 500, 531, 539, 547, 600, 617, 662, 690, 706, 770, 879, 969, 1086, 1112, 1127, 1136, 1189, 1202, 1223, 1257, 1318, 1378, 2925 | 0.753 |
| EP3 | 15, 26, 38, 51, 68, 71, 83, 99, 125, 143, 194, 218, 337, 346, 363, 452, 516, 543, 557, 585, 633, 649, 727, 816, 900, 1065, 1106, 1145, 1191, 1211, 1244, 1256, 1384, 1613, 3814, 3918 | 0.752 |
| EP4 | 7, 13, 34, 43, 54, 83, 126, 150, 174, 196, 215, 336, 346, 364, 452, 517, 546, 558, 585, 633, 649, 731, 816, 905, 1073, 1110, 1134, 1193, 1210, 1254, 1270, 1383, 2924 | 0.752 |
| EP5 | 18, 38, 40, 54, 70, 81, 102, 113, 130, 178, 207, 225, 316, 339, 354, 426, 501, 529, 576, 602, 635, 680, 723, 824, 892, 1045, 1128, 1139, 1197, 1219, 1241, 1262, 1383, 1605, 3814, 3919 | 0.752 |
| EP6 | 15, 17, 41, 43, 51, 83, 130, 159, 173, 206, 228, 316, 339, 354, 426, 502, 530, 577, 603, 636, 682, 728, 828, 893, 1056, 1128, 1134, 1201, 1216, 1252, 1270, 1383, 2912 | 0.752 |

**Table S3**. Relative energies (Er) of main species for reactions 5–10 at the B3LYP/6-311++G(d,p), CCSD(T)//B3LYP/6-311++G(d,p) levels (in kcal/mol) with the ZPE correction and *T*_1_ diagnostic values obtained at the CCSD(T)//B3LYP/6-311++G(d,p) level.

| Species | ZPE | *T*_1_ | B3LYP | CCSD(T)//B3LYP |  |
| --- | --- | --- | --- | --- | --- |
| R1 + OH | 0.074571 | 0.013, 0.010 | 0.0 | 0.0 |  |
| ER1 | 0.075608 | 0.013 | －1.44 | －4.26 |  |
| TS1 | 0.070819 | 0.016 | 2.35 | 5.89 |  |
| EP1 | 0.075408 | 0.014 | －18.54 | －16.81 |  |
| P1+ H_2_O | 0.073735 | 0.014, 0.011 | －16.19 | －5.95 |  |
| R1 + Cl | 0.06612 | 0.013, 0.006 | 0.0 | 0.0 |  |
| TS2 | 0.058034 | 0.014 | 8.12 | 10.17 |  |
| EP2 | 0.059816 | 0.014 | －2.25 | －0.09 |  |
| P1 + HCl | 0.059116 | 0.014, 0.006 | －1.87 | 9.09 |  |
| R2a + OH | 0.062139 | 0.013, 0.010 | 0.0 | 0.0 |  |
| ER3 | 0.063116 | 0.013 | －2.03 | －1.80 |  |
| TS3 | 0.058796 | 0.017 | 0.24 | 5.97 |  |
| EP3 | 0.062692 | 0.014 | －16.01 | －14.48 |  |
| P2a + H_2_O | 0.061799 | 0.014, 0.011 | －14.77 | －11.80 |  |
| R2a + Cl | 0.053688 | 0.013, 0.006 | 0.0 | 0.0 |  |
| ER4 | 0.053839 | 0.013 | －0.53 | －0.11 |  |
| TS4 | 0.046202 | 0.015 | 3.11 | 8.29 |  |
| EP4 | 0.048074 | 0.014 | －0.73 | 1.96 |  |
| P2a + HCl | 0.04718 | 0.014, 0.0056 | －0.45 | 3.24 |  |
| R2b + OH | 0.062143 | 0.014, 0.010 | 0.0 | 0.0 |  |
| ER5 | 0.063131 | 0.013 | －1.22 | －1.98 |  |
| TS5 | 0.058977 | 0.017 | 0.09 | 5.43 |  |
| EP5 | 0.062937 | 0.014 | －15.77 | －14.57 |  |
| P2b + H_2_O | 0.061826 | 0.014, 0.011 | －14.77 | －11.63 |  |
| R2b + Cl | 0.053692 | 0.014, 0.006 | 0.0 | 0.0 | |
| ER6 | 0.053857 | 0.013 | －0.54 | －0.17 | |
| TS6 | 0.04622 | 0.015 | 3.05 | 7.94 | |
| EP6 | 0.04813 | 0.014 | －0.70 | 0.88 | |
| P2b + HCl | 0.047207 | 0.014, 0.0056 | －0.46 | 3.41 | |

**Table S4**. Reaction enthalpies ($\Delta H_{r,298}^{\theta}$) and reaction Gibbs free energies ($\Delta G_{r,298}^{\theta}$) for reactions 7–10 at the B3LYP/6-311++G(d,p), and CCSD(T)//B3LYP/6-311++G(d,p) levels (kcal/mol) with the ZPE or TZPE (thermal corrections to enthalpy or Gibbs free energies) corrections.

|  | B3LYP | CCSD(T)//B3LYP |
| --- | --- | --- |
| $\Delta H_{r,298}^{\theta}$ | | |
| CF_3_OCF_2_CF_2_H (a) + OH → CF_3_OCF_2_CF_2_ (a) + H_2_O | －14.44 | －11.48 |
| CF_3_OCF_2_CF_2_H (a) + Cl → CF_3_OCF_2_CF_2_ (a) + HCl | 0.17 | 3.86 |
| CF_3_OCF_2_CF_2_H (b) + OH → CF_3_OCF_2_CF_2_ (b) + H_2_O | －14.46 | －11.32 |
| CF_3_OCF_2_CF_2_H (b) + Cl → CF_3_OCF_2_CF_2_ (b) + HCl | 0.15 | 4.01 |
| $\Delta G_{r,298}^{\theta}$ |  | |
| CF_3_OCF_2_CF_2_H (a) + OH → CF_3_OCF_2_CF_2_ (a) + H_2_O | －15.81 | －12.84 |
| CF_3_OCF_2_CF_2_H (a) + Cl → CF_3_OCF_2_CF_2_ (a) + HCl | －2.43 | 1.26 |
| CF_3_OCF_2_CF_2_H (b) + OH → CF_3_OCF_2_CF_2_ (b) + H_2_O | －15.66 | －12.52 |
| CF_3_OCF_2_CF_2_H (b) + Cl → CF_3_OCF_2_CF_2_ (b) + HCl | －2.29 | 1.58 |

**Table S5. Experimental enthalpies of formation () values involved in the reactions of 16–27.**

| Species |  |
| --- | --- |
| CH_3_ | 34.82 kcal/mol ^a^ |
| CH_4_ | –17.89 kcal/mol ^a^ |
| CF_3_OCHF_2_ | –312.20 kcal/mol ^b^ |
| CF_3_ | –112.40 kcal/mol ^c^ |
| CH_3_F | –56.80 kcal/mo1^a^ |
| CH_2_F_2_ | –108.20 kcal/mol ^d^ |
| CHF_3_ | –166.60 kcal/mol ^a^ |
| CH_3_CF_3_ | –178.94 kcal/mol ^a^ |
| CHFCF_3_ | –166.50 kcal/mol ^a^ |
| CF_3_CF_3_ | –321.20 kcal/mol ^a^ |
| CF_3_CH_2_CF_3_ | –366.50 kcal/mol ^c^ |
| CH_3_OCH_3_ | –43.99 kcal/mol ^a^ |

^a^Obtained from Ref. ^9^

^b^Obtained from Ref. ^11^

^c^Obtained from Ref. ^12^

^d^Obtained from Ref. ^14^

**Table S6.** Calculated frequencies (in cm^-1^) for the reactants, products, transition states, and complexes for the oxidation and decomposition pathways of alkoxy radicals at the B3LYP/6-311++G(d,p) level.

| species | B3LYP/6-311++G(d,p) |
| --- | --- |
| CF_3_OCF_2_CF_2_O | 35, 47, 85, 123, 202, 227, 282, 314, 334, 359, 394, 502, 514, 541, 573, 603, 636, 663, 699, 826, 878, 934, 1070, 1101, 1126, 1166, 1207, 1228, 1245, 1284 |
| (CF_3_)_2_C(O)OCF_3_ | 36, 50, 59, 70, 142, 148, 200, 214, 264, 289, 296, 309, 325, 351, 444, 454, 532, 534, 544, 546, 602, 607, 666, 704, 744, 799, 817, 865, 1049, 1055, 1059, 1153, 1181, 1184, 1199, 1231, 1236, 1246, 1253 |
| CF_3_OCF_2_CFO | 30, 40, 83, 127, 219, 244, 324, 350, 365, 417, 509, 529, 610, 630, 664, 727, 760, 824, 894, 1064, 1108, 1133, 1199, 1232, 1247, 1309, 1949 |
| CF_3_COOCF_3_ | 24, 55, 92, 123, 208, 245, 319, 363, 428, 430, 516, 543, 572, 610, 657, 745, 769, 862, 886, 1090, 1158, 1159, 1215, 1217, 1250, 1314, 1897 |
| CF_3_COCF_3_ | 38, 39, 143, 190, 254, 270, 310, 365, 463, 499, 523, 532, 622, 709, 761, 781, 956, 1119, 1164, 1182, 1222, 1244, 1303, 1876 |
| CF_3_COCF_2_ | 25, 63, 173, 355, 372, 460, 516, 587, 610, 640, 719, 898, 1057, 1144, 1179, 1228, 1243, 1282 |
| O_2_(^3^P) | 1633 |
| CF_3_O | 229, 402, 568, 585, 609, 882, 1130, 1175, 1239 |
| CF_3_ | 502, 503, 691, 1062, 1222, 1223 |
| COF_2_ | 575, 614, 772, 955, 1201, 1974 |
| FO_2_ | 224, 417, 1636 |
| TS1-CC | 395*i*, 29, 44, 65, 150, 171, 205, 247, 274, 354, 380, 396, 459, 527, 553, 576, 599, 625, 688, 773, 851, 873, 1007, 1078, 1123, 1206, 1222, 1271, 1295, 1533 |
| TS1-F | 265*i*,34, 46, 78, 119, 138, 209, 225, 281, 324, 353, 362, 426, 501, 528, 609, 611, 653, 667, 724, 826, 898, 1064, 1121, 1148, 1201, 1236, 1254, 1310, 1690 |
| TS1-O2 | 573*i*, 7, 32, 34, 55, 77, 87, 128, 143, 193, 227, 261, 286, 319, 340, 354, 373, 443, 507, 526, 598, 609, 642, 665, 713, 823, 889, 1054, 1122, 1133, 1184, 1223, 1233, 1282, 1553, 1665 |
| TS2-CC | 329*i*,20, 48, 55, 66, 121, 139, 170, 200, 219, 234, 284, 312, 351, 393, 438, 467, 529, 535, 538, 557, 576, 612, 657, 718, 748, 816, 876, 989, 1037, 1159, 1164, 1191, 1204, 1226, 1237, 1274, 1287, 1534 |
| TS2-CO | 414*i*, 11, 31, 51, 60, 115, 132, 145, 188, 264, 269, 279, 312, 356, 383, 417, 463, 496, 524, 544, 565, 597, 613, 646, 664, 709, 767, 883, 950, 1118, 1126, 1177, 1178, 1190, 1200, 1225, 1229, 1251, 1517 |

**Table S7.** Frequencies, IR intensities, and radiative forcing are used in calculation of GWP of CF_3_OCH(CF_3_)_2_

| Frequencies (cm^-1^) | A_k_ (IR intensities) (km/mol) | A_k_ (IR intensities) (cm/molecule) | Radiative forcing per Unit Cross Section 10^15^ W m^-2^(cm^-1^)^-1^(cm^2^ molecule^-1^)^-1 Ref.43^ | Radiative forcing (W/m^2^ppbv) | Total Radiative forcing (W/m^2^ppbv) |
| --- | --- | --- | --- | --- | --- |
| 17.6066 | 0.0027 | 4.49E-22 | 0.0145 | 6.50E-09 | 6.32E-01 |
| 31.6554 | 0.0184 | 3.06E-21 | 0.0213 | 6.51E-08 |
| 58.209 | 0.1301 | 2.16E-20 | 0.0676 | 1.46E-06 |
| 73.1498 | 0.0423 | 7.03E-21 | 0.0724 | 5.09E-07 |
| 143.2886 | 0.9305 | 1.55E-19 | 0.255 | 3.94E-05 |
| 160.1369 | 2.6628 | 4.42E-19 | 0.665 | 2.94E-04 |
| 209.3777 | 0.7526 | 1.25E-19 | 0.484 | 6.05E-05 |
| 220.0093 | 0.8411 | 1.40E-19 | 0.42 | 5.87E-05 |
| 288.9705 | 0.0587 | 9.75E-21 | 0.776 | 7.57E-06 |
| 308.0074 | 0.0947 | 1.57E-20 | 0.825 | 1.30E-05 |
| 315.5016 | 0.7411 | 1.23E-19 | 0.732 | 9.01E-05 |
| 339.0935 | 0.1086 | 1.80E-20 | 0.862 | 1.56E-05 |
| 450.5325 | 0.0281 | 4.67E-21 | 1.26 | 5.88E-06 |
| 472.031 | 5.2796 | 8.77E-19 | 1.46 | 1.28E-03 |
| 517.8066 | 7.1907 | 1.19E-18 | 1.97 | 2.35E-03 |
| 530.3715 | 2.7351 | 4.54E-19 | 2.2 | 1.00E-03 |
| 544.5159 | 1.1917 | 1.98E-19 | 1.96 | 3.88E-04 |
| 547.5811 | 3.244 | 5.39E-19 | 2.21 | 1.19E-03 |
| 608.8232 | 0 | 0.00E+00 | 1.36 | 0.00E+00 |
| 648.3866 | 3.2002 | 5.32E-19 | 0.0573 | 3.05E-05 |
| 667.6423 | 27.3536 | 4.54E-18 | 0.0496 | 2.25E-04 |
| 683.8062 | 50.6166 | 8.41E-18 | 0.0777 | 6.53E-04 |
| 737.604 | 7.8891 | 1.31E-18 | 1.44 | 1.89E-03 |
| 862.8926 | 44.8717 | 7.45E-18 | 3.17 | 2.36E-02 |
| 894.3944 | 39.6339 | 6.58E-18 | 3.1 | 2.04E-02 |
| 898.3969 | 5.2906 | 8.79E-19 | 3.01 | 2.65E-03 |
| 1093.433 | 81.733 | 1.36E-17 | 2.18 | 2.96E-02 |
| 1112.616 | 49.0026 | 8.14E-18 | 2.07 | 1.68E-02 |
| 1130.128 | 106.7133 | 1.77E-17 | 1.79 | 3.17E-02 |
| 1165.493 | 302.9418 | 5.03E-17 | 1.52 | 7.65E-02 |
| 1178.071 | 204.4849 | 3.40E-17 | 1.52 | 5.16E-02 |
| 1199.223 | 511.8283 | 8.50E-17 | 1.67 | 1.42E-01 |
| 1230.259 | 764.5824 | 1.27E-16 | 1.26 | 1.60E-01 |
| 1245.531 | 152.6921 | 2.54E-17 | 0.815 | 2.07E-02 |
| 1265.537 | 459.2398 | 7.63E-17 | 0.455 | 3.47E-02 |
| 1293.391 | 135.9195 | 2.26E-17 | 0.359 | 8.11E-03 |
| 1369.026 | 126.3567 | 2.10E-17 | 0.18 | 3.78E-03 |
| 1406.951 | 22.7959 | 3.79E-18 | 0.098 | 3.71E-04 |

**Table S8.** Frequencies, IR intensities, and radiative forcing are used in calculation of GWP of CF_3_OCF_2_CF_2_H.

| Frequencies (cm^-1^) | A_k_ (IR intensities) (km/mol) | A_k_ (IR intensities) (cm/molecule) | Radiative Forcing per Unit Cross Section 10^15^ W m^-2^(cm^-1^)^-1^(cm^2^ molecule^-1^)^-1 Ref.43^ | Radiative forcing (W/m^2^ppbv) | Total Radiative forcing (W/m^2^ppbv) |
| --- | --- | --- | --- | --- | --- |
| 34.937 | 0.0449 | 7.46E-21 | 0.213 | 1.59E-06 | 6.35E-01 |
| 63.4058 | 1.4635 | 2.43E-19 | 0.0676 | 1.64E-05 |
| 86.7425 | 0.291 | 4.83E-20 | 0.112 | 5.41E-06 |
| 131.1423 | 0.6312 | 1.05E-19 | 0.202 | 2.12E-05 |
| 201.8548 | 1.2949 | 2.15E-19 | 0.337 | 7.25E-05 |
| 216.2454 | 2.3606 | 3.92E-19 | 0.42 | 1.65E-04 |
| 334.7316 | 0.1727 | 2.87E-20 | 0.856 | 2.46E-05 |
| 345.1005 | 0.2658 | 4.42E-20 | 0.836 | 3.69E-05 |
| 363.6624 | 0.2201 | 3.66E-20 | 1.02 | 3.73E-05 |
| 450.5962 | 0.1034 | 1.72E-20 | 1.26 | 2.16E-05 |
| 519.5813 | 5.4534 | 9.06E-19 | 1.97 | 1.78E-03 |
| 544.3548 | 6.8529 | 1.14E-18 | 1.96 | 2.23E-03 |
| 560.238 | 8.2372 | 1.37E-18 | 2.01 | 2.75E-03 |
| 585.4493 | 63.0933 | 1.05E-17 | 1.19 | 1.25E-02 |
| 627.6035 | 3.8076 | 6.32E-19 | 0.365 | 2.31E-04 |
| 644.5559 | 9.4399 | 1.57E-18 | 0.0777 | 1.22E-04 |
| 754.9111 | 44.9362 | 7.46E-18 | 2.37 | 1.77E-02 |
| 820.7727 | 3.2188 | 5.35E-19 | 3.19 | 1.71E-03 |
| 956.6916 | 7.0933 | 1.18E-18 | 2.81 | 3.31E-03 |
| 1099.444 | 308.1235 | 5.12E-17 | 2.1 | 1.07E-01 |
| 1112.589 | 19.8228 | 3.29E-18 | 2.07 | 6.82E-03 |
| 1125.419 | 194.2041 | 3.23E-17 | 1.79 | 5.77E-02 |
| 1135.979 | 227.3613 | 3.78E-17 | 1.88 | 7.10E-02 |
| 1196.733 | 630.3078 | 1.05E-16 | 1.67 | 1.75E-01 |
| 1214.288 | 435.6307 | 7.24E-17 | 1.23 | 8.90E-02 |
| 1248.852 | 606.806 | 1.01E-16 | 0.815 | 8.22E-02 |
| 1294.716 | 48.7883 | 8.10E-18 | 0.359 | 2.91E-03 |

**
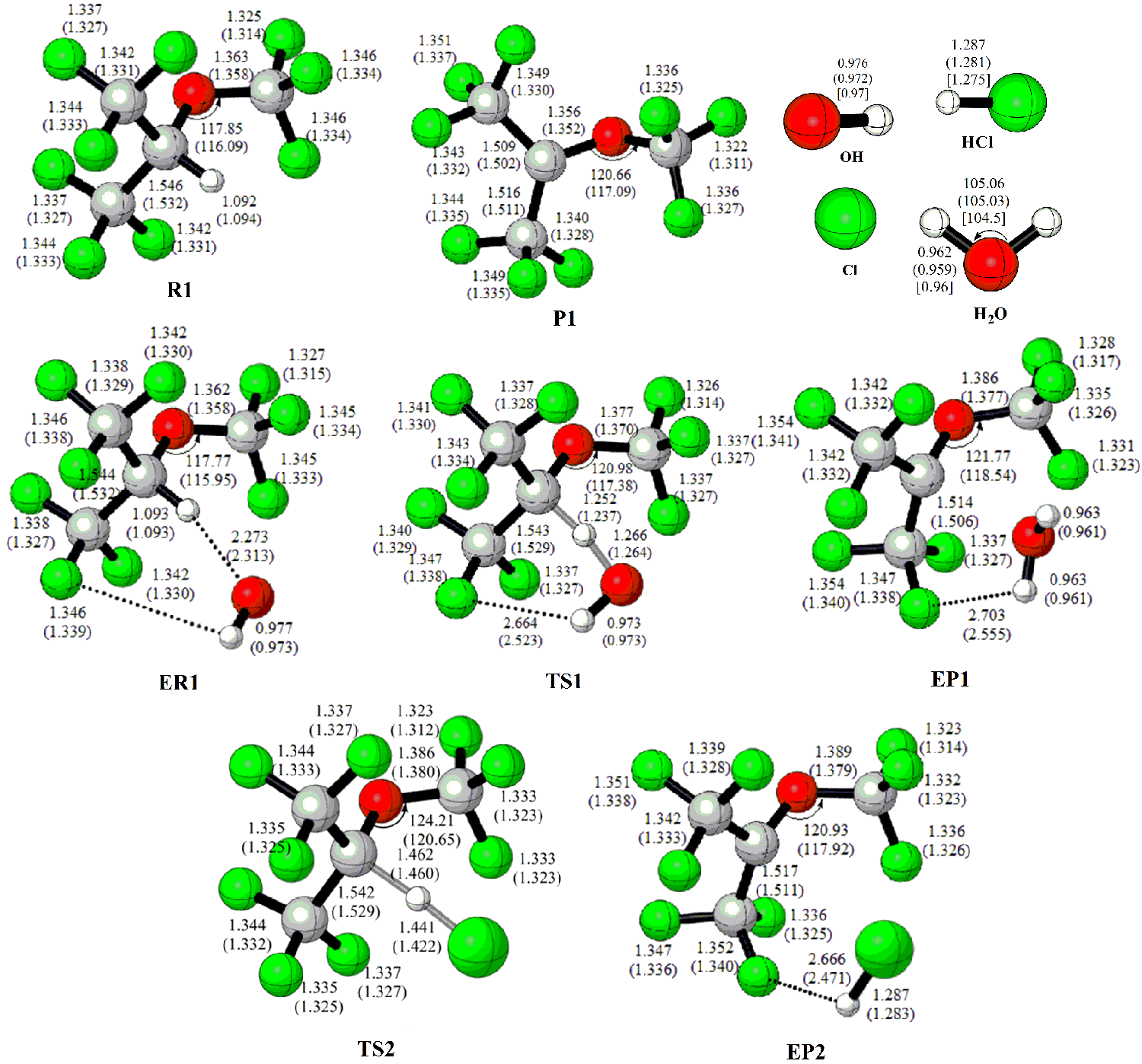
**

**Figure S1. (*Continued*)**


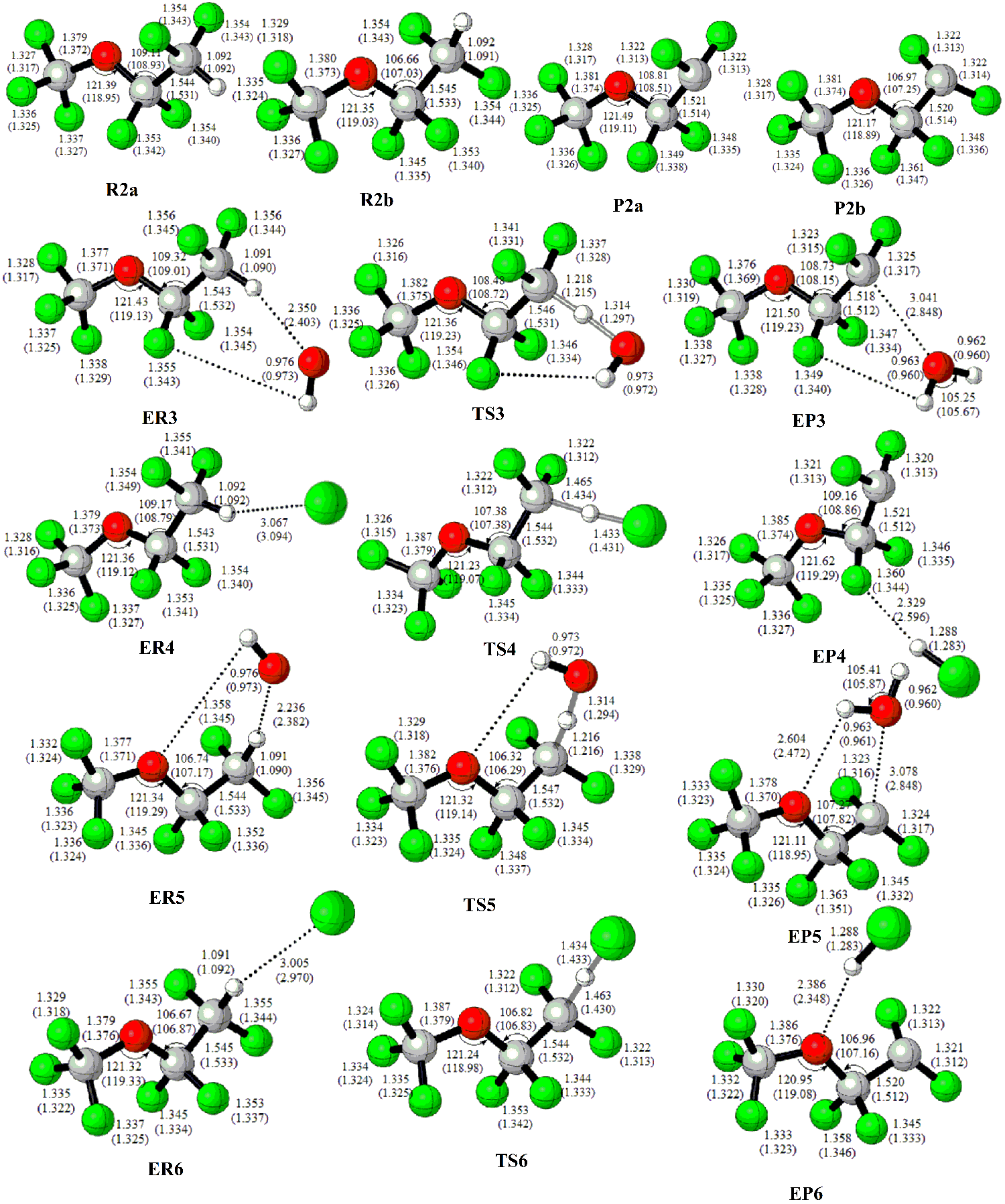


**Figure S1.** Optimized geometries of the reactants, products, transition states, and complexes at the B3LYP/6-311++G(d,p), M06-2X/6-311++G(d,p) (in parentheses) levels for CF_3_OCH(CF_3_)_2_/CF_3_OCF_2_CF_2_H (a, b) + OH/Cl reactions, and the limited experimental values (in square brackets). Bond lengths are in angstroms and angles in degrees.


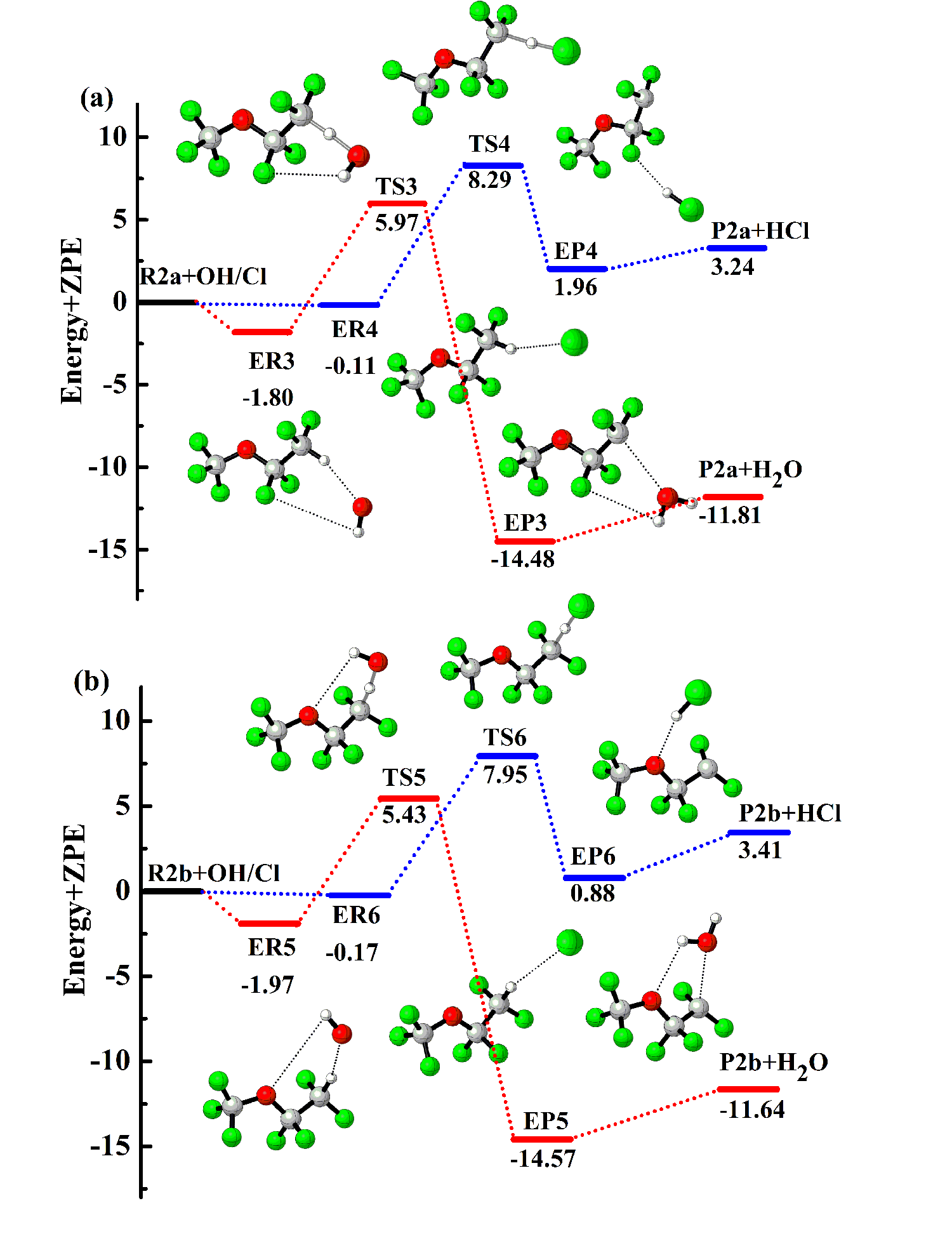


**Figure S2.** Schematic potential energy surface for reaction CF_3_OCF_2_CF_2_H (a, b) + OH/Cl. The relative energies (in kcal/mol) are calculated at the CCSD(T)//B3LYP/6-311++G(d,p) + ZPE level.


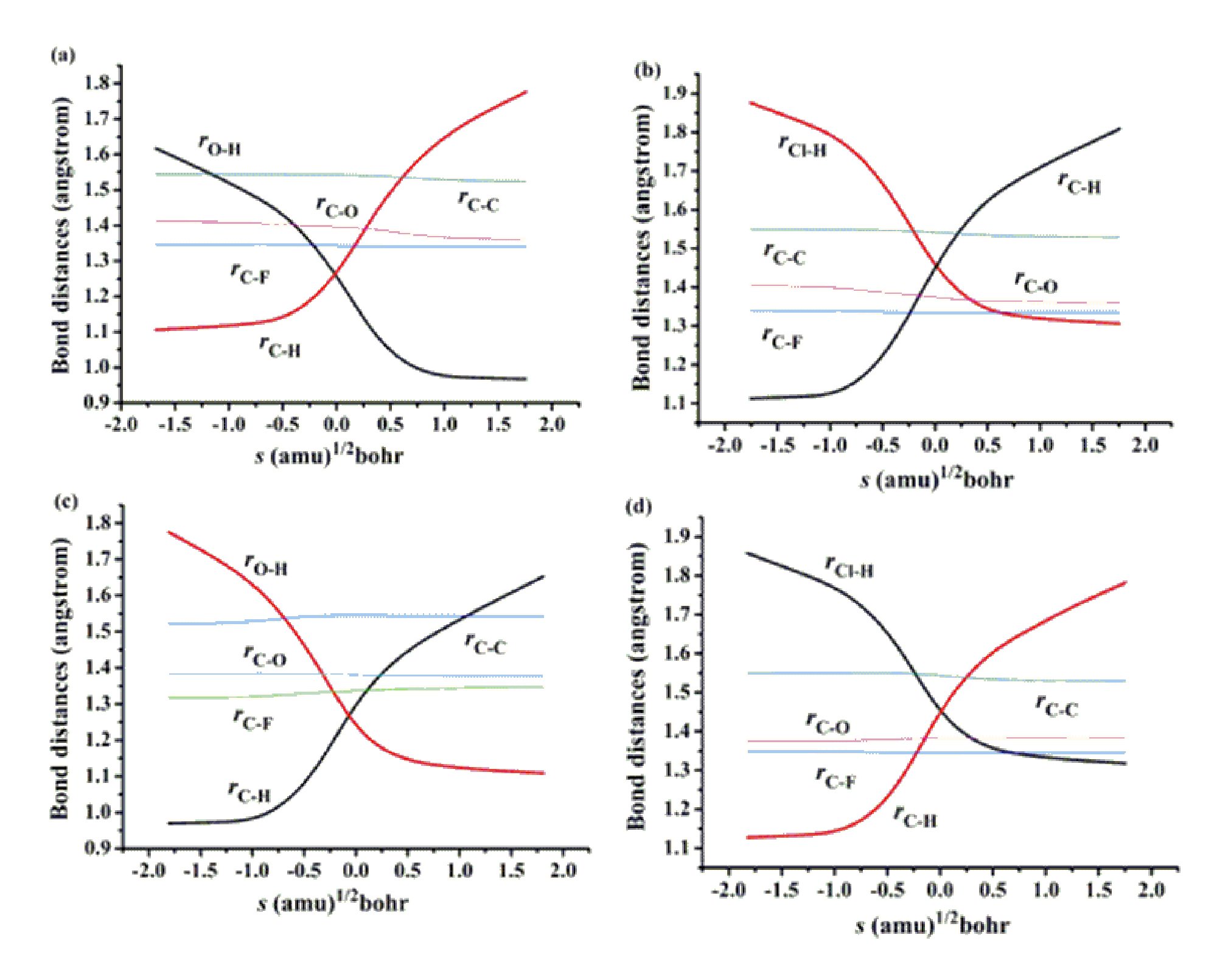


**Figure S3.** Changes of the bond distances (in angstroms) for the reactions 5 (a), 6 (b), 7 (c), and 8 (d).

**
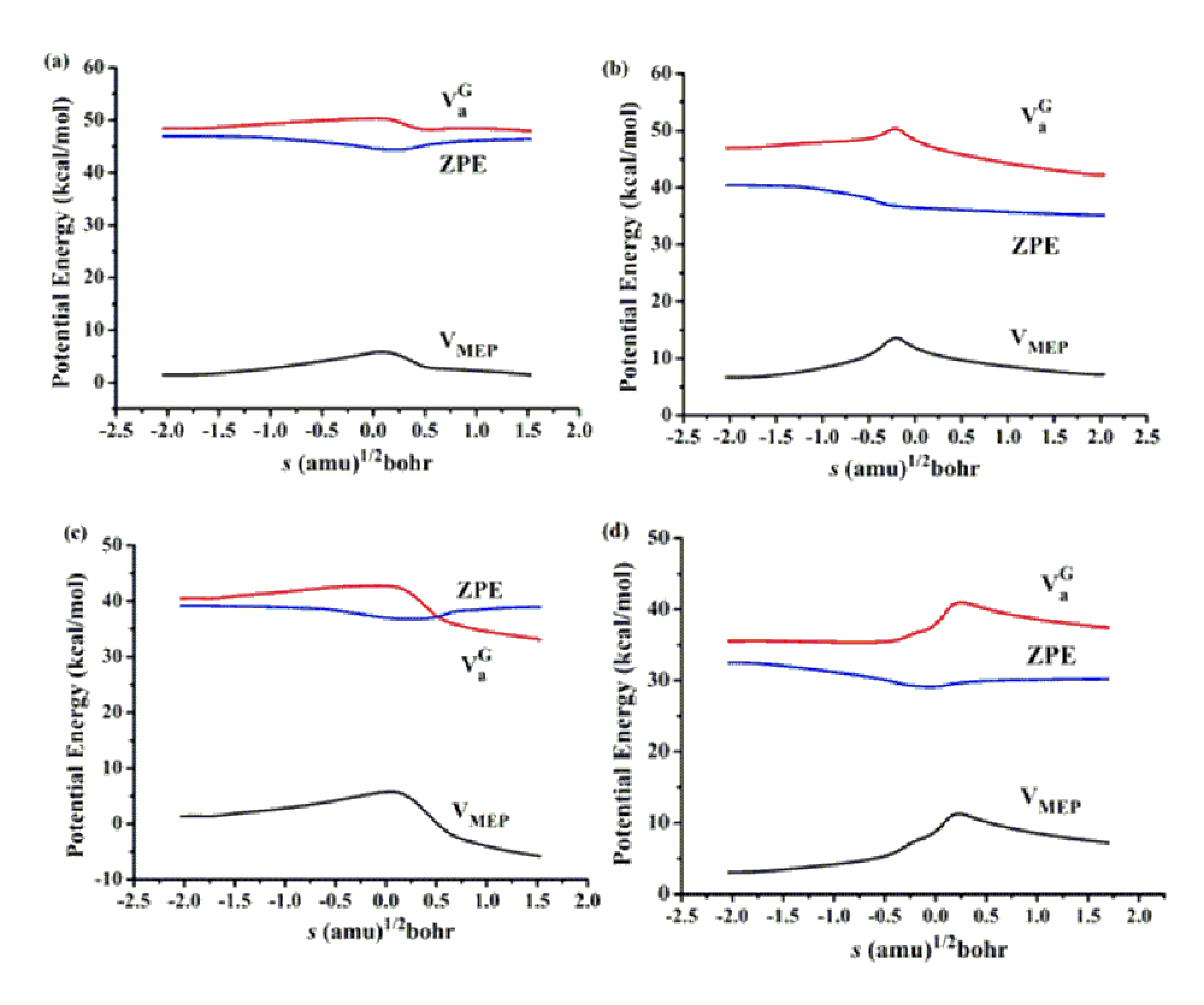
**

**Figure S4.** Classical potential energy curve (*V*_MEP_), ground-state vibrational adiabatic energy curve (*V*), and zero-point energy curve (ZPE) as functions of *s* (amu)^1/2^bohr at the CCSD(T)//B3LYP/6-311++G(d,p) level for the reactions 5 (a), 6 (b), 7 (c), and 8 (d).

**
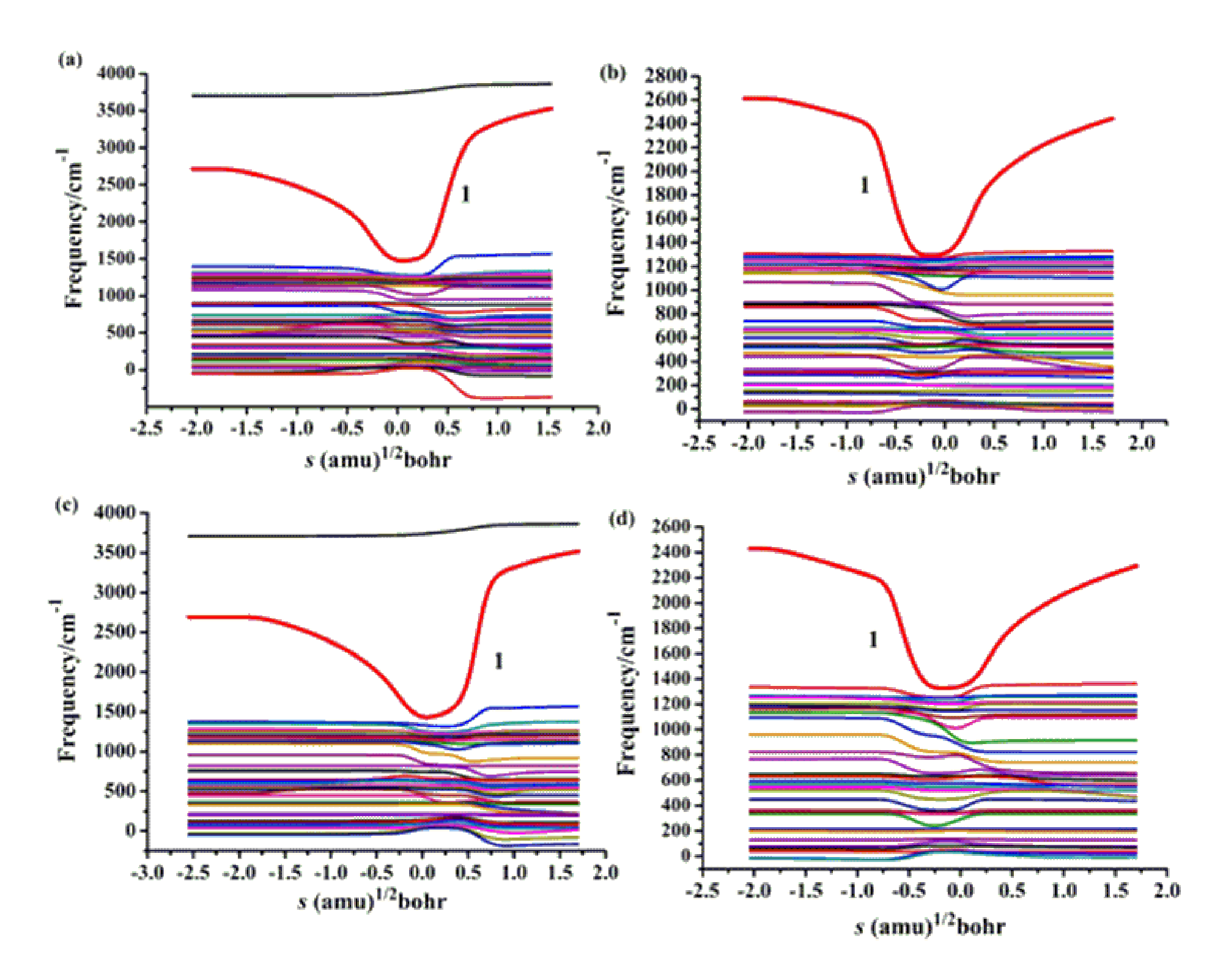
**

**Figure S5.** Changes of generalized normal-mode vibrational frequencies as functions of *s* (amu)^1/2^bohr at the CCSD(T)//B3LYP/6-311++G(d,p) level for the reactions 5 via TS1 (a), 6 via TS2 (b), 7 via TS3 (c), and 8 via TS4 (d).

**
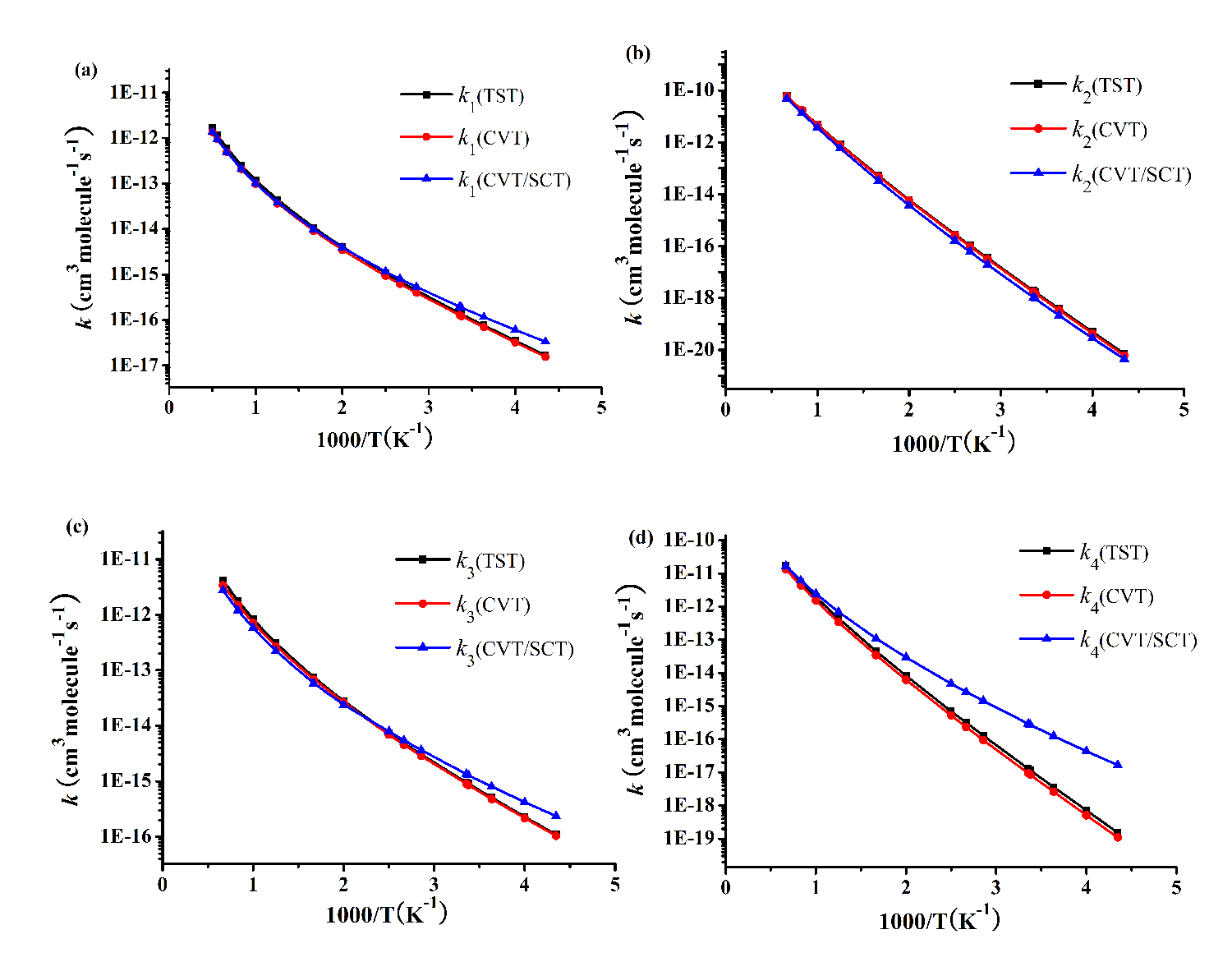
**

**Figure S6.** Calculated TST, CVT, and CVT/SCT rate constants as functions of 1000/*T* for reactions 5 (a), 6 (b), 7 (c), and 8 (d) in the temperature range of 230–1500 K.


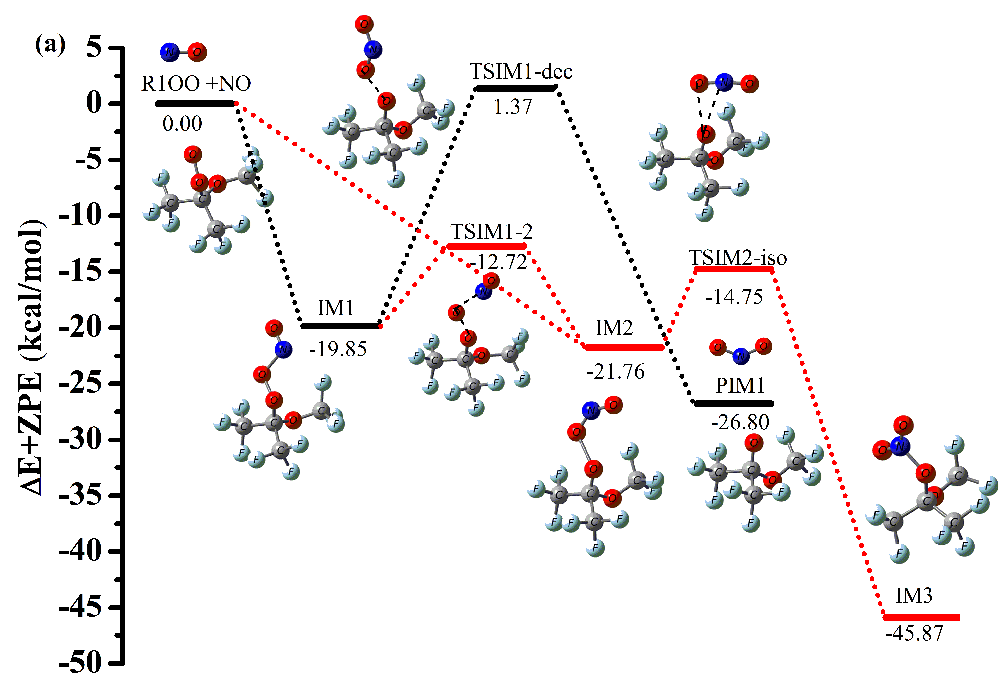


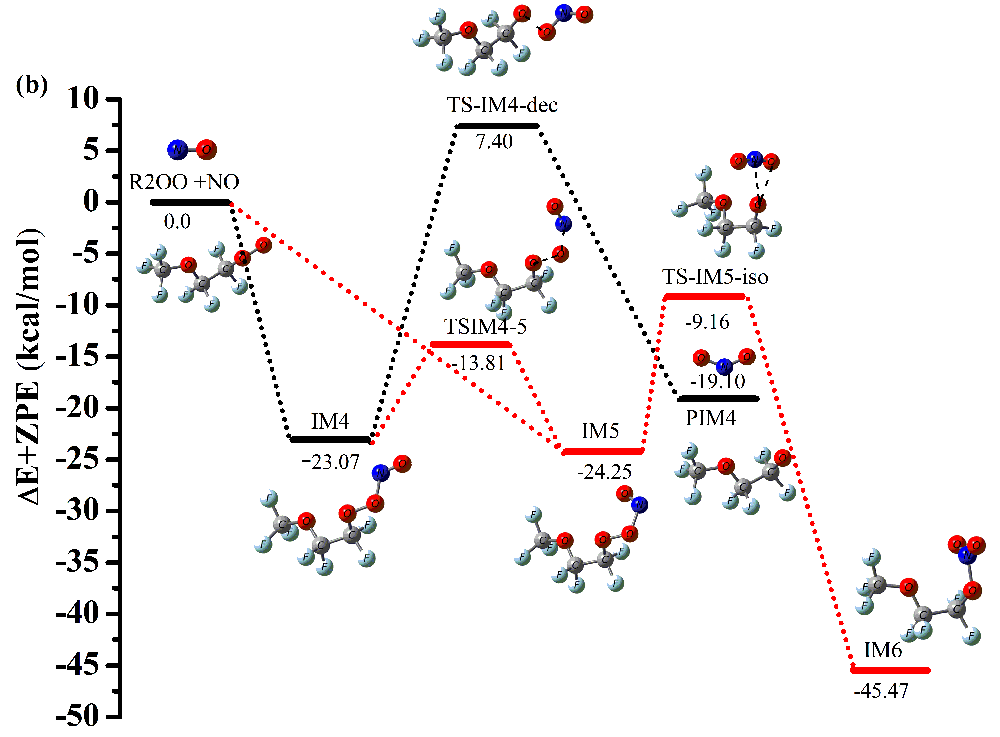


**Figure S7.** Schematic potential energy surface for reaction CF_3_OC(OO•)(CF_3_) _2_ + NO (a) and CF_3_OCF_2_CF_2_OO• + NO (b). The relative energies (in kcal/mol) are calculated at the B3LYP/6-311++G(d,p) level.


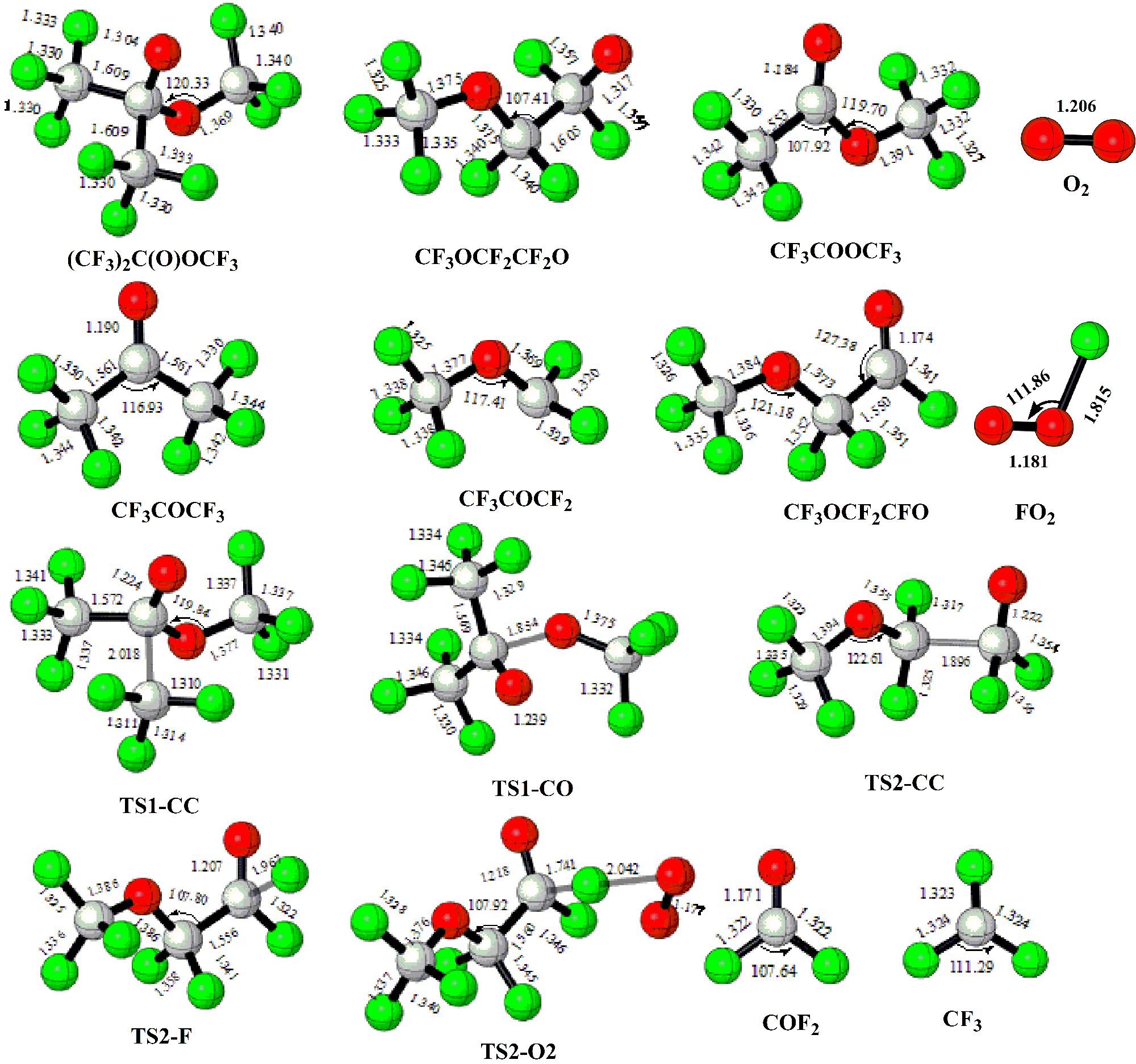


**Figure S8.** Optimized geometries of reactants, products, and transition states for the oxidation and decomposition channels of alkoxy radicals at the B3LYP/6-311++G(d,p) level.


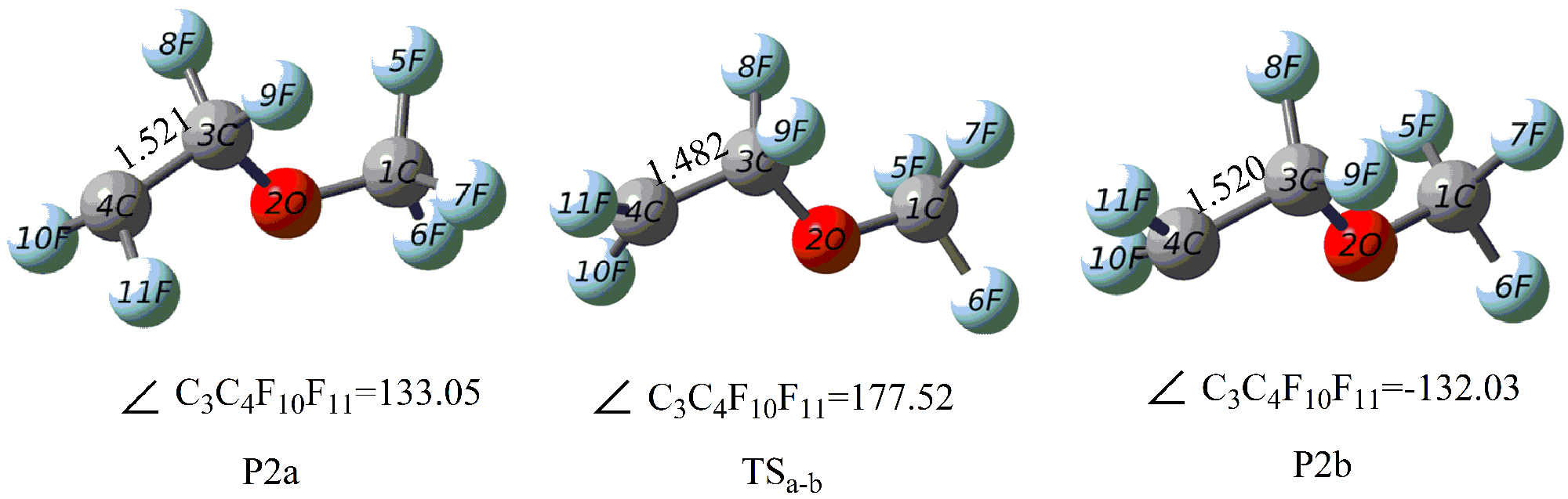


**Figure S9.** Optimized geometries of the CF_3_OCF_2_CF_2_H (a,b) and TS_a-b_ at the B3LYP/6-311++G(d,p) level. Bond lengths are in angstroms and angles in degrees.
